# Polycation-π Interactions Are a Driving Force for Molecular Recognition by an Intrinsically Disordered Oncoprotein Family

**DOI:** 10.1371/journal.pcbi.1003239

**Published:** 2013-09-26

**Authors:** Jianhui Song, Sheung Chun Ng, Peter Tompa, Kevin A. W. Lee, Hue Sun Chan

**Affiliations:** 1 Departments of Biochemistry, Molecular Genetics, and Physics, University of Toronto, Toronto, Ontario, Canada; 2 Division of Life Science, Hong Kong University of Science and Technology, Clear Water Bay, Hong Kong S.A.R., China; 3 VIB Department of Structural Biology, Vrije Universiteit Brussel, Brussels, Belgium; 4 Institute of Enzymology, Hungarian Academy of Sciences, Budapest, Hungary; Fudan University, China

## Abstract

Molecular recognition by intrinsically disordered proteins (IDPs) commonly involves specific localized contacts and target-induced disorder to order transitions. However, some IDPs remain disordered in the bound state, a phenomenon coined “fuzziness”, often characterized by IDP polyvalency, sequence-insensitivity and a dynamic ensemble of disordered bound-state conformations. Besides the above general features, specific biophysical models for fuzzy interactions are mostly lacking. The transcriptional activation domain of the Ewing's Sarcoma oncoprotein family (EAD) is an IDP that exhibits many features of fuzziness, with multiple EAD aromatic side chains driving molecular recognition. Considering the prevalent role of cation-π interactions at various protein-protein interfaces, we hypothesized that EAD-target binding involves polycation- π contacts between a disordered EAD and basic residues on the target. Herein we evaluated the polycation-π hypothesis via functional and theoretical interrogation of EAD variants. The experimental effects of a range of EAD sequence variations, including aromatic number, aromatic density and charge perturbations, all support the cation-π model. Moreover, the activity trends observed are well captured by a coarse-grained EAD chain model and a corresponding analytical model based on interaction between EAD aromatics and surface cations of a generic globular target. EAD-target binding, in the context of pathological Ewing's Sarcoma oncoproteins, is thus seen to be driven by a balance between EAD conformational entropy and favorable EAD-target cation-π contacts. Such a highly versatile mode of molecular recognition offers a general conceptual framework for promiscuous target recognition by polyvalent IDPs.

## Author Summary

Understanding how proteins recognize each other is central to deciphering the inner workings of living things and for biomedical research. It has long been known that the sequence of a protein, which is a string of different amino acids, can dictate how a protein molecule folds into a well-defined shape required for biological tasks. Many folded proteins recognize and bind with each other by a tight geometric fit similar to that between a lock and its key. Recently, however, it has become clear that some proteins function as a flexible string, in constant motion, without forming a stable shape. Understanding how such “disordered” proteins work is challenging. To gain insight, we studied a disordered protein region that causes a large family of human cancers. Employing an innovative combination of experimental and theoretical techniques, we describe a new mode of protein interaction based on multiple simple contacts between one type of amino acid (aromatic) in the disordered protein and another type (positively charged) on the partner protein. Because this mechanism also underlies the ability of the disordered protein to cause cancer, further investigation of this unprecedented mode of protein-protein interaction may open up new avenues for cancer therapy.

## Introduction

Understanding the sequence-function relationship of a protein and how it might malfunction is central to biomedical research. While many proteins function in their folded states, recently it became clear that intrinsically disordered proteins (IDPs) also play key functional roles [1], [2] in transcription, translation and cell cycle regulation that, when altered, frequently lead to cancer [3]. Indeed, ∼70% of proteins implicated in cancer are predicted to have significant disordered regions [3], [4]. Molecular recognition by IDPs typically involves target-induced folding. Intriguingly, however, certain IDPs engage in protein-protein interaction without coupled folding and binding [5] such that the IDP remains disordered even when bound to a globular target. This phenomenon has been termed “fuzziness” [6] and is characterised by IDP polyvalency, sequence-insensitivity and lack of strict geometric complementarity for binding [6]. Important examples of fuzziness include transcription factors [7], linker histones [8], prion-like proteins [9] and Sic1-Cdc4 in yeast [10].

To gain insight into “fuzzy” interactions, we have studied the Ewing's Sarcoma (EWS)-activation domain (EAD) in the TET family of RNA-binding proteins [11] and Ewing's family of oncoproteins (EFPs). EAD is a ∼280 residue polyvalent IDP comprised mainly of a degenerate repeat motif SYGQQS. Studies of EAD have mostly focused on its role in naturally occurring EFPs in which it is fused to various transcription factor partners. EFPs are potent EAD-dependent transcriptional activators, resulting in distinct phenotypes of the associated Ewing's family of tumors [12], [13] which are largely dictated by the DNA-binding domain of the EWS fusion partner. Progress in understanding EAD has been hindered by its IDP properties [14] and a general lack of biophysical/biochemical insights [15]. Another barrier is the paucity of information regarding cognate EAD-interacting proteins. Because native EWS interacts with a highly complex array of proteins at a network hub [16], [17] or potentially as a scaffold protein [18], it is probable that EAD has numerous partners.

Functional studies of EFPs have provided a foundation for understanding sequence-function relationship of EAD. Most notably, the transcriptional and oncogenic activity of EAD is conferred by multiple tyrosine (Y) residues due to their aromaticity but not hydrophobicity [14]. EAD function is also markedly sequence-insensitive [14], although a permissive overall composition is apparently required. This type of interaction shares features with other systems that exploit polyvalent IDP phosphorylation, as in autoinhibition of CFTR [19], auto-regulation of Ets-1 transcription factor [20], [21] and interaction of Cdk inhibitor Sic1 with its E3 ubiquitin ligase Cdc4 [10]. Sic1 has nine low-affinity Cdc4-binding sites and a threshold number of phosphorylated sites induces highly cooperative “polyelectrostatic” binding of Sic1 to a single positively charged pocket in Cdc4 [10], [22]–[24]. Similarly, EAD activity requires cooperative action of multiple aromatic moieties in a disordered structure [14], [25], though it does not require phosphorylation. Thus molecular recognition by EAD was coined “polyaromatic” [26]. However, the physical basis for polyaromatic EAD function has not been elucidated.

In light of the versatile roles of cation-π interactions in protein folding and protein-protein interactions [27]–[37], we hypothesized that a major contribution to molecular recognition by EAD (within EFPs) is the attraction between numerous unconstrained aromatic residues (π's) on the EAD and basic residues (cations) on the target. We tested this idea experimentally and also theoretically in molecular simulations that are based on cation-π contacts between the EAD and a generic folded target. We found broad agreement between EAD functionality and simulated EAD binding. Thus our findings strongly support the polycation-π model and suggest that similar mechanisms might also be exploited by other IDPs.

## Results

### Rationale of the investigation

In view of the abundance of aromatic residues in EAD (38 Ys in the native EAD) and the significant strength of cation-π interactions [38], we posit cation-π as a highly plausible and probably most significant type of EAD-target contacts. This leaves open the possibility of additional contributions but these are likely to be secondary [14]. To probe the nature of EAD-target interactions we designed EAD mutants with different numbers, placements and types of aromatic residues in conjunction with EAD charge variations (Fig. S1). In vivo transcriptional activity of EAD mutants was compared, relatively, with computational predictions of binding probability assuming the polycation-π hypothesis. In the absence of specific knowledge about EAD targets, a generic globular target with appropriate surface charge was used for simulations to provide a minimalist physical model for the proposed interactions.

### Functional effect of Y-dosage is consistent with simulated EAD-target binding via cation-π interactions

**Figure 1:**
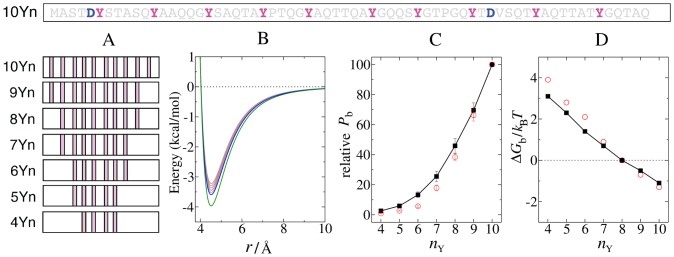
Initial test of the polycation-π model: Y number dependence. (A) EAD peptide sequences. The complete sequence for 10Yn is shown at the top with Ys (magenta) and Ds (blue) highlighted. The Yn series (4Yn–10Yn) are derived from 10Yn and contain the indicated numbers and positions of Ys such that the Y density is the same for all proteins. (B) Total interaction energy between a cation-aromatic pair in the model including the 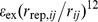 excluded-volume term in Eq. **(S1)**, where 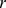 is the distance 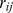 between the cation and the aromatic residue. The well depths for cation-Y (blue curve) and cation-W (green curve) are taken to be 3.58 and 4.0 kcal/mol, respectively. The brown curves provide a range of plausible well depths between 3.21 and 3.51 kcal/mol for cation-F (Text S1). (C) Effect of Y number 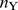
on transactivation and simulated binding. Relative transcriptional activity of the EAD peptides (open red circles) was determined under sub-saturating conditions (Methods and Text S1) relative to 10Yn activity (arbitrarily set to 100). Red error bars for the experimental data indicate SEM. The relative 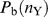 values (filled black squares) are normalized by the 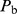 for 10Yn [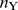 = 10, actual simulated (absolute) 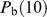 = 0.43]. The black error bars mark standard deviations among ten independent simulations. (D) Model binding free energy 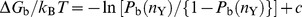 (filled black squares; see Text S1) for the same set of EAD sequences. As an example, the constant *c* is chosen such that 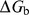 = 0 at 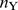 = 8. 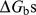 for different *c* values correspond to different EAD concentrations (see analytical model). Also shown is a free-energy-like quantity 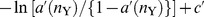
derived from experiment (open red circles) where 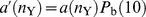, 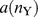 is the relative activity in (C), and 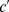 is chosen so that this quantity coincide with 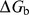 = 0 at 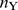 = 8 to facilitate comparison.

The intact EAD spans ∼280 highly repetitive residues but such a long sequence is not particularly amenable to mutagenesis and is also quite impractical for computational studies. This hurdle can be overcome by exploiting small EAD regions (∼40 residues) that faithfully mimic the salient features of the intact EAD and whose transcriptional activity (transactivation) can be readily tested using a multisite reporter [14], [25]. To establish a framework for experiments, we began by functionally testing a 66-residue polypeptide (10Yn) with ten Y residues, which is closely related to part of the native EAD, and the corresponding series of mutants (4Yn–9Yn) varying only in Y number (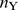) but retaining the same Y density (Fig. 1A). Transactivation was quantified by a well-established transient assay in Jeg3 cells with EAD sequences fused to the DNA-binding domain of zta protein and a zta reporter plasmid (pZ7luc) [39] (Methods). As for other EAD sequences studied before [25], transactivation rises in a nonlinear manner with 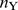 (Fig. 1C, red circles), demonstrating that multiple Ys act together in a cooperative manner.

To assess the polycation-π idea, we constructed a coarse-grained chain simulation model that embodies the hypothesis. The EAD is represented by a flexible 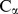 chain and a generic globular target protein is modeled as a sphere with surface charge distribution (Fig. S2A,B) resembling that of the RNA polymerase II subunits Rpb4/Rpb7 (PDB id: 2C35; Fig. S2C), which was reported to bind to EAD [40], [41]. Binding is driven by EAD-target cation-π contacts (Fig. S2D), the interaction energies (Fig. 1B and Fig. S2E) of which are consistent with published estimates of cation-π potentials of mean force in aqueous environments, with attractive well depths ≈−3.0 to −5.5 kcal/mol [32], [33], [38]. In accordance with PDB data [30], [32], contacts between one cation and multiple aromatics or between one aromatic and multiple cations are allowed; but the orientation dependence [42] and nonadditivity [43] of cation-π interactions are neglected. Because EAD-target cation-π interactions are suggested to be highly dynamic with bound EAD remaining disordered, we included an average solvation effect [38] rather than considering the discrete water configurations that impact on cation-π interactions [44]. Our model also incorporates electrostatic and intra-EAD hydrophobic effects (Fig. S2F) by using potential functions similar to those developed for coarse-grained protein folding simulations [45], [46]; but EAD-target hydrophobic interactions were not considered because of insufficient knowledge about the real target. Binding probability (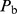) was determined using Monte Carlo sampling (see Methods and Supporting Text S1 for details).

Fig. 1C shows that the simulated *P*_b_s rationalize the functional data regarding the effect of Y (aromatic) number. A similar agreement with model simulation was also observed for the activities of a set of previously studied EAD sequences (Fig. S3). Noting that the EAD-zta proteins used for determining activity are dimers whereas EAD monomers were used in our simulations, we also verified that the EAD monomer and dimer 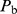 values have a similar Y-number dependence (Fig. S4), indicating that EAD monomer simulations are adequate for capturing behavioral trends of the corresponding EAD dimers.

We emphasize that the experimental-theoretical comparisons in Fig. 1 and subsequent figures are between relative experimental activities and relative 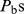. The model binding free energy 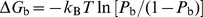, where 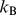 is Boltzmann constant and *T* is absolute temperature (Fig. 1D), is dependent upon the effective EAD concentration (see below). However, the latter is unknown experimentally and our simple model does not account for every physical interaction between the real EAD and its target. Thus, it is not meaningful to compare absolute 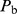 against absolute experimental activity. Nonetheless, by assuming that putative unknown factors affect different EAD sequences similarly (Text S1), one may compare the differences in simulated 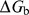 for various EAD sequences with the corresponding differences in EAD activity. Doing so yielded a good agreement between experiment and theory for the 4Yn–10Yn sequences (Fig. 1D), lending support to the polycation-π hypothesis.

### An analytical model of polycation-π mediated IDP binding to a folded target

To better understand how EAD binding might be affected by various assumptions about the target and multisite IDP binding in general, we developed a simple analytical model to complement the chain simulations. Briefly, our analytical model considers an IDP chain of *n* contour length units with 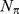 equally spaced aromatic residues that are *k* units apart, and a target with 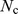 cations. When the IDP is distant from the partner, it can adopt 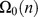 conformations with any residue fixed in space; that residue in turn can access a volume *V* (i.e., the IDP concentration is 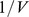). Binding is favored by an energy 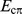 (<0) for each IDP-target cation-π contact. A bound IDP has ≥1 such contact, with 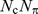 possible pairings for the first contact. Because the volume accessible to the first contacting residue is reduced from *V* to a small “capture” volume 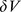 and the number of IDP conformations is reduced from 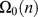 to a smaller 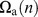 because of IDP-target excluded volume, it follows that the change in free energy upon forming the first contact is 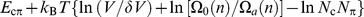. For 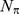
>1, further cation-π contacts can lead to IDP loops of various lengths 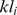 (where 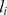 = 1, 2, …; Fig. S5A) spanning a variety of distances 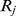 between different cations on the target (Fig. S5B). If 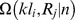 is the number of IDP conformations of length *n* with such a loop and 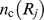 is the number of instances of 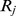, the free energy of binding 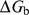 is approximately given by:
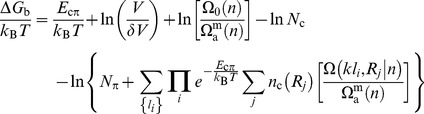
(1)where we have used the number of conformations 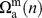 with a mid-chain attachment for 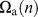, neglecting the small variation in 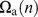 that depends on the attaching point (Text S1); thus 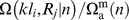 is the conformational reduction factor for forming an IDP loop. 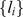 in 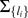 represents all 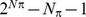 possible sets of ≥2 aromatic residues that can contact the target (resulting in 1 to 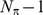 loops). The 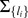 term vanishes when 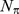 = 1. 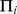 is over the different loops for a given set of contacting residues. We assumed that the loops are independent and neglected the excluded volume repulsion among them. Exact enumeration of self-avoiding lattice flights [47] (Figs. S5C–E, S6, Supporting Tables S1, S2, S3) and extrapolations of such data (Fig. S7) were applied to estimate the conformational entropy terms involving 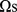 in Eq. **(1)**. Further details of the model are provided in Text S1.

**Figure 2:**
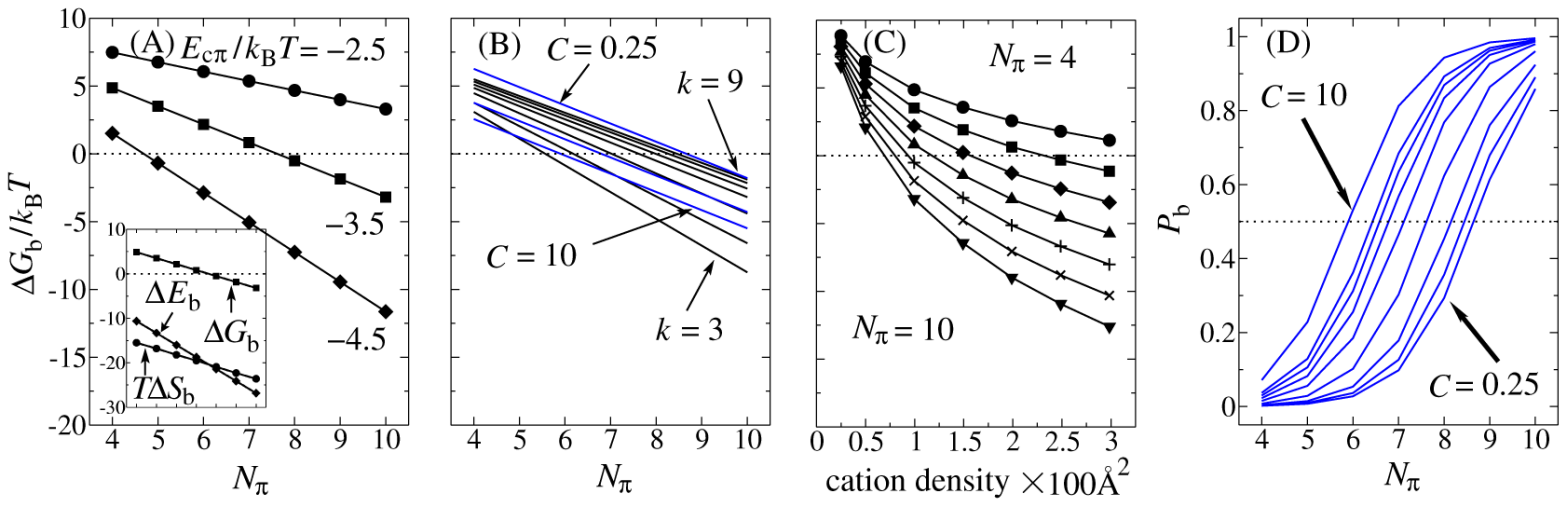
IDP-target binding in the analytical model. To match the chain simulation model, we used 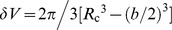 = 438.0 Å^3^, where *b* = 3.8 Å is the 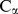
–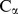 virtual bond length and 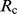 = 6 Å is the capture radius for a cation-π contact in the chain model. (A) The IDP's chain length *n* = 66, with *k* = 6 (corresponding to the sequences in Fig. 1). 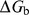 was computed for different 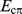 values. 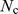 = 32 for the target and *V* = (600 Å)^3^ as in the simulations [hence 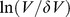 = 13.1]. Inset: The energy (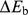) and entropy (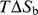) components of 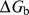 for 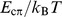 = −3.5. Results in (B–D) are also for 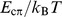 = −3.5. (B) Effects of *k* and *V* on binding; 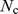 = 32; 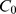 = 1/(600 Å)^3^ is used as a reference IDP concentration. The black curves show 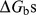 at 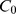 for hypothetical sequences with *k* = 9, 8, 7, 6, 5, 4, and 3 (from top to bottom), *n* = 66 for *k*≤6 and *n* = 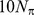 for *k*≥7. The blue curves are for the *k* = 6 sequences for three IDP concentrations 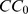 with *C* = 0.25, 3.0, and 10.0 (from top to bottom). (C) 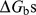 for *k* = 6 sequences at *C* = 1 on different targets of the same size with different 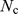 = 8, 16, 32, 48, 64, and 80 (from left to right; see Text S1 and Fig. S5B). (D) 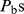 of the *k* = 6 sequences at different IDP concentrations *C* = 10.0, 5.0, 4.0, 3.0, 2.0, 1.0, 0.5, 0.33, and 0.25 (from top to bottom).

Salient features of the analytical model are shown in Fig. 2. An essentially linear dependence of 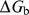 on 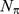 is seen (Fig. 2A) as for the simulation results (Fig. 1D). As expected, a stronger (more negative) 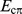 leads to tighter binding (more negative 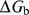). The binding equilibrium is governed by a balance between favorable cation-π contacts on one hand and translational and conformational entropy on the other (Fig. 2A, *inset*). Binding increases with aromatic density 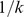, IDP concentration *C* (Fig. 2B,D; *C*∼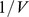), and target cation density (Fig. 2C). Fig. 2A shows that the 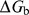 trend for 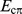
≈−3.5*k*_B_ *T* in our analytical model matches approximately the behavior of 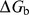 in the chain simulation in Fig. 1D. This value of 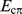
≈−2.1 kcal/mol (for *T* = 300 K used in this study) is comparable but weaker than the average pairwise cation-Y energy ≈−3.3 kcal/mol we determined from our simulation using a cation-Y potential energy well depth of ≈3.6 kcal/mol (Fig. 1B). This discrepancy is not unexpected because excluded volume effects among the loops are neglected in Eq. **(1)**, resulting in an overestimation of binding probability. Nonetheless, the overall trend exhibited by the chain simulation model is well reflected by the analytical model.

### Efficacy of different cation-π strengths and intramolecular competition by EAD cations supports the model

**Figure 3:**
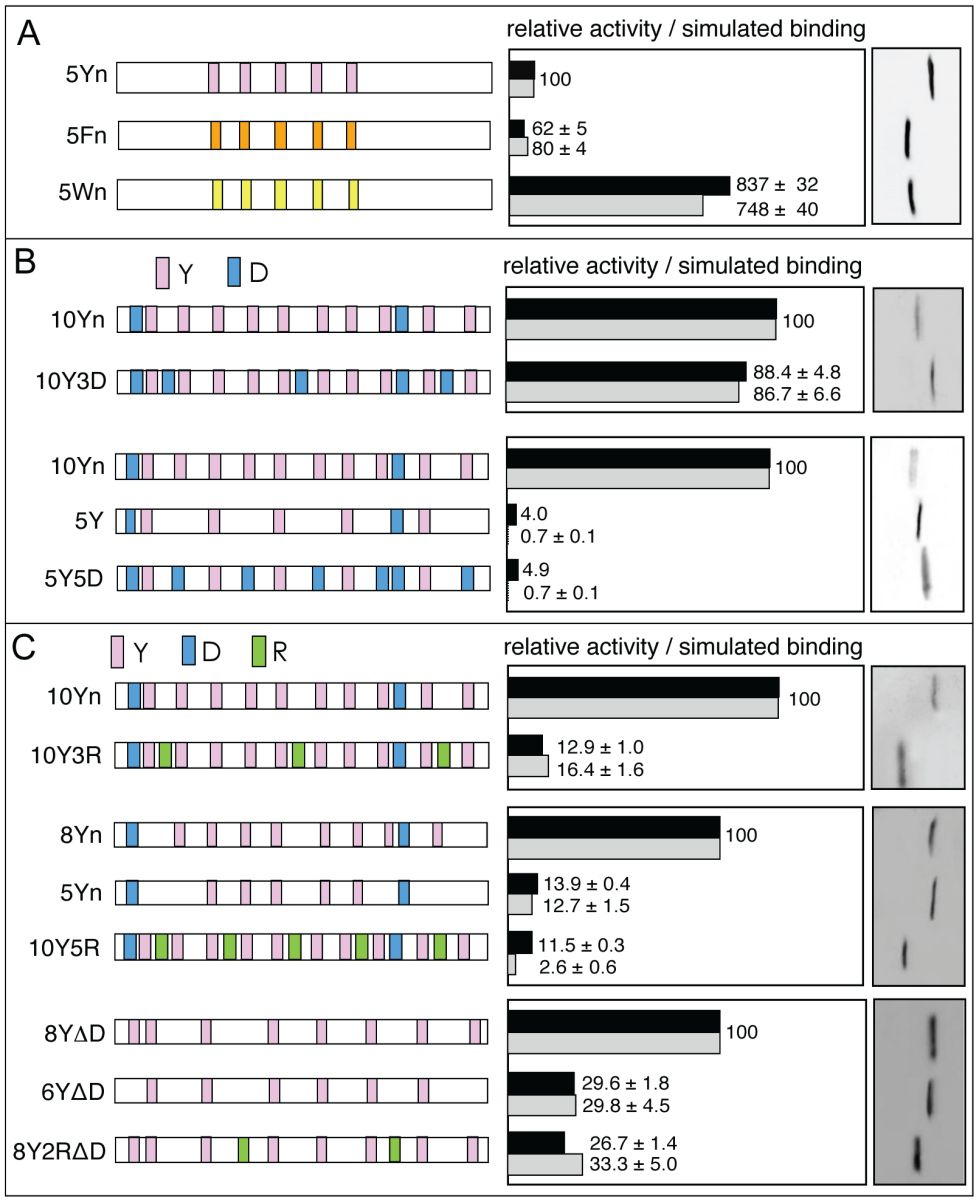
Further testing of the polycation-π model. Designed mutant EADs (left) were tested for transcriptional activity and simulated binding. Full peptide sequences are given in Fig. S1. Y residues for all peptides are shown in magenta as in Fig. 1 and the key residues are similarly depicted. Protein expression levels were determined by Western blot analysis of epitope-tagged activator proteins in extracts from transfected cells using KT3 antibody (right). The histograms show percentage experimental activities (black) and simulated 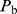 (grey) relative to that of the first sequence (100%) in each experiment. Estimated errors for simulated 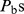 are standard deviations from ten independent simulations. (A) Efficacy of different aromatic moieties. All Ys in 5Yn (Fig. 1A) were replaced by W (yellow) or F (orange). The variation of well depth for cation-F (Fig. 1B) entails a range of relative 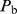 from 24% to 80% and the latter is plotted here. (B) Effect of adding anions (Asp, shown in blue). (C) Effect of adding cations (Arg, shown in green).

In addition to accounting for Y-number dependence (Fig. 1), the cation-π hypothesis also rationalizes EAD activity of mutants with Y substituted by phenylalanine (F) or tryptophan (W). Statistical analysis of PDB structures [32] and quantum calculations [48] have indicated that the cation-Y and cation-F strengths are similar, with F slightly weaker [48], but cation-W is significantly stronger (Text S1). Consistent with this trend, Fig. 3A shows that the experimental activity of 5Fn is slightly lower than that of 5Yn [25], but the activity of 5Wn is ∼8 fold that of 5Yn. Simulated 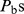 for these sequences using the corresponding cation-π energies in Fig. 1B mirror these experimental observation, lending further credence to the polycation-π hypothesis.

We next investigated the effect of altering EAD charge. First, we changed anion composition by introducing aspartic acid (D) residues (Fig. 3B). Adding 3 Ds to 10Yn (10Y3D) or adding 5 Ds to the minimally active 5Y (5Y5D) barely changes activity. The fact that anion additions do not enhance EAD activity rules out favorable contacts between EAD anions and target cations as a major driving force for binding. Second, we changed cation composition by introducing arginine (R) residues (Fig. 3C). Inasmuch as the 66-residue EAD peptides are flexible as posited by our chain simulation model, the inserted Rs would allow intra-EAD cation-π contacts and thus reduce activity by competition. Fig. 3C shows that an EAD with 10 Ys and 5 Rs (10Y5R) is indeed much less active than one with only 8 Ys and zero Rs (8Yn) and is comparable with a protein containing only 5 Ys. Similarly, 8Y2RΔD (containing 8 Ys and 2 Rs) is comparable with 6YΔD (6 Ys and zero Rs) and both EADs are approximately 3-fold less active than 8YΔD (8Ys and zero Rs). Apparently, the addition of R residues within the EAD functionally counteracts Ys in an essentially one-to-one manner. This finding is highly suggestive of Y-R contacts between EAD and real target proteins and thereby strongly supports the cation-π hypothesis.

The relative simulated 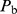 values broadly capture the activity trends for charge variations (Fig. 3B,C). Quantitative agreement between simulation and experiment is seen for 10Y3D, 5Yn, 6YΔD, and 8Y2RΔD. Simulation also accounts for the near-irrelevance of anion number for 5Y and 5Y5D activities (Fig. 3B). Simulations did however slightly overestimate the decrease in activity caused either by reduction of Y number from 10 to 5 (Fig. 3B, compare 10Yn with 5Y or 5Y5D) or by introduction of cations into 10Yn (Fig. 3C, compare 8Yn with 10Y5R). The average EAD-target electrostatic energy is essentially neutral or very slightly repulsive in our model (+0.2 kcal/mol). Because of the dominance of cation-π over electrostatic interactions (Fig. S2E,F), 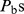 of 10Yn and 10Y3D are very similar; but there is some EAD-target electrostatic repulsion due to the anions on 10Y3D, resulting in a slightly weaker average EAD-target cation-π energy for 10Y3D compared with 10Yn (−22.2 vs −23.4 kcal/mol). Intra-EAD cation-π interactions in the unbound state are strong in R-containing mutants, amounting on average to −31.9 kcal/mol for 10Y3R and −67.6 kcal/mol for 10Y5R and are slightly weaker in the bound state (−24.9 and −63.6 kcal/mol respectively). The favorable EAD-target cation-π energy acquired upon binding is −18.5 kcal/mol for 10Y3R and −7.0 kcal/mol for 10Y5R on average, indicating that the weaker binding of 10Y5R is caused by increased competition from intra-EAD cation-π interactions due to the larger number of Rs present.

### Interplay between number of cation-π contacts and EAD conformational entropy determines activity

**Figure 4:**
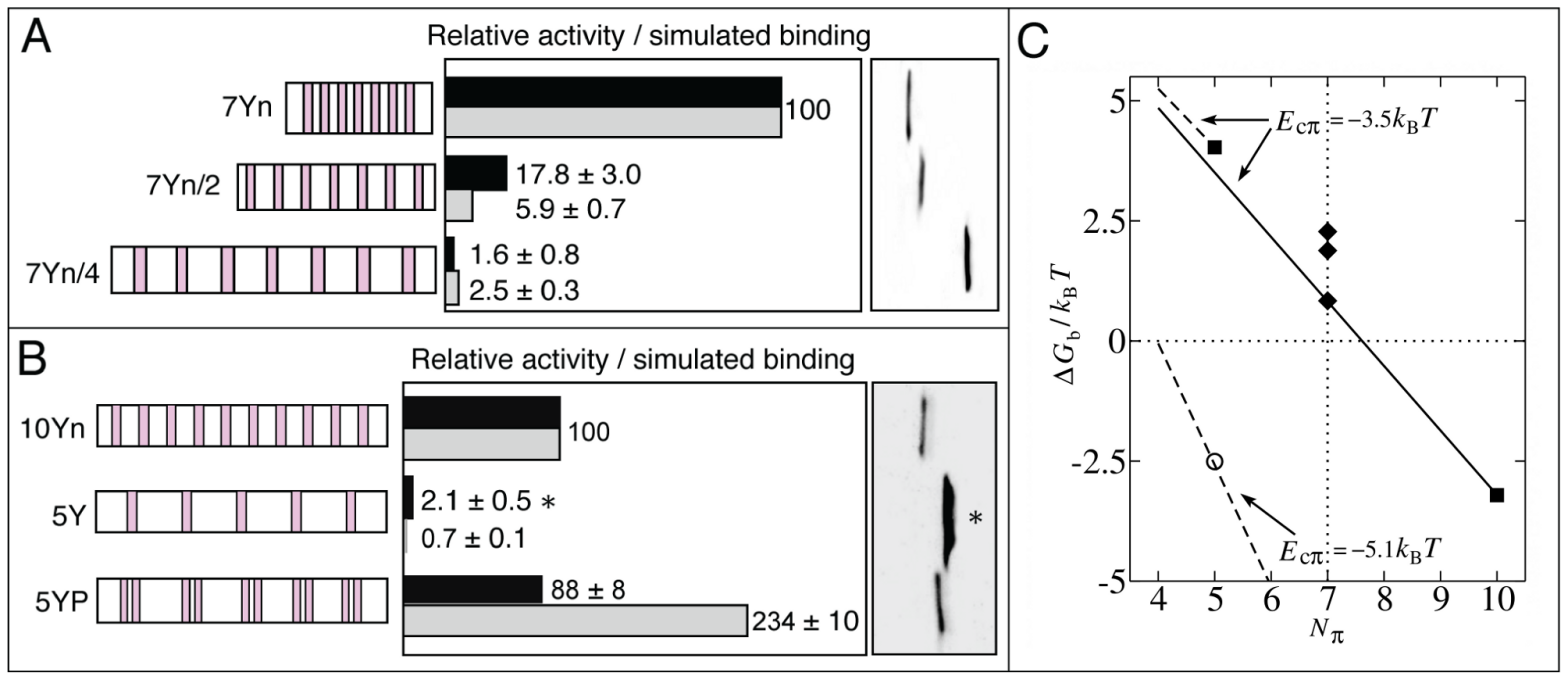
Effect of Y density and distribution on EAD activity. (A, B) The EAD peptides (left) were tested for relative transactivation (black) and simulated 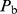 (grey), shown in the same style as in Fig. 3. (A) 7Yn (see Fig. 1A) with Y density denoted normal (n or *k* = 6) was compared with 7Yn/2 (Y density ∼1/2 of 7Yn, *k* = 12) and 7Yn/4 (Y density ∼1/4 that of 7Yn, *k* = 24). The actual simulated 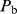 for 7Yn is 0.11. (B) 10Yn (see Fig. 1A; *k* = 6) was compared with 5Y (*k* = 12) and the sequence 5YP which has 5 pairs of sequentially adjacent Ys. The asterisk indicates that 5Y activity is overstated due to relatively higher expression of 5Y protein. (C) Analysis using our analytical model. All 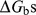 were for 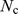 = 32, *C* = 1, and 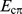 = −3.5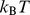 except the data point plotted as open circle (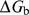 = −2.6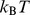) was for 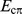 = −5.1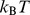. The solid line shows results for *k* = 6 and *n* = 66. The upper and lower dashed lines provide results for *k* = 12 with chain lengths *n* = 66 and *n* = 71 respectively. The diamonds show results (from bottom to top) for 7Yn, 7Yn/2, and 7Yn/4 in (A), which have chain lengths *n* = 66, 86, and 156 respectively. To facilitate comparison with the 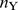 = 7 data in (A), 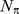 = 7 is marked by the vertical dotted line. The squares show results for 5Y (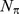 = 5; 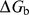 = 4.0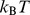) and 10Yn (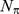 = 10; 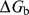 = −3.2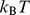) in (B), both with *n* = 66. As discussed in Text S1, the model represented by the open circle may be applied to 5YP in (B) with −5.1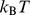 as the interaction energy between a cation and two adjacent aromatic residues.

As shown in Fig. 2B, the polycation-π hypothesis envisions that EAD activity depends on both aromatic number and density. We tested this prediction using EAD sequences with constant Y number (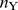 = 7) but different Y densities (1/*k* values). The data in Fig. 4A show both experimental activity and simulated binding diminish with decreasing Y density 1/*k*. This trend is consistent with the analytical model results for these sequences (Fig. 4C, diamonds), although the analytical model predicts a less pronounced decrease.

Is EAD activity affected by altering the sequence positions (distribution) of the Ys while maintaining overall density and total Y number? In Fig. 4B, sequence 5YP has a total of 10 Ys arranged as 5 pairs separated by ∼12 residues and has transcriptional activity similar to 10Yn (88%) and in excess of 40-fold more active than 5Y. Simulations (Fig. 4B) and the analytical model (Fig. 4C, squares and circle) generally reflect the activity trend but overestimate 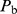 for 5YP compared with 10Yn. This mismatch probably results from the simplifying model assumption that each individual cation-π contact for two adjacent Ys interacting simultaneously with the same cation is equivalent to an isolated cation-π contact, whereas in reality adjacent Ys would each have somewhat weaker interaction due to steric hindrance by each other and the orientation dependence of cation-π interactions ([42] and Text S1). Taken together, these results indicate that Y density influences EAD activity but that Y distribution is not crucial. Physically, weaker binding at lower Y densities arises from at least two conformational entropy effects that result from longer loops between cation-π contacts: formation of an individual longer loop is entropically more costly than a shorter loop [47], and excluded volume interference between longer loops is also more severe. Both effects disadvantage longer loops and disfavor binding of EAD sequences with lower Y densities.

## Discussion

### A distinctive fuzzy protein-protein interaction

**Figure 5:**
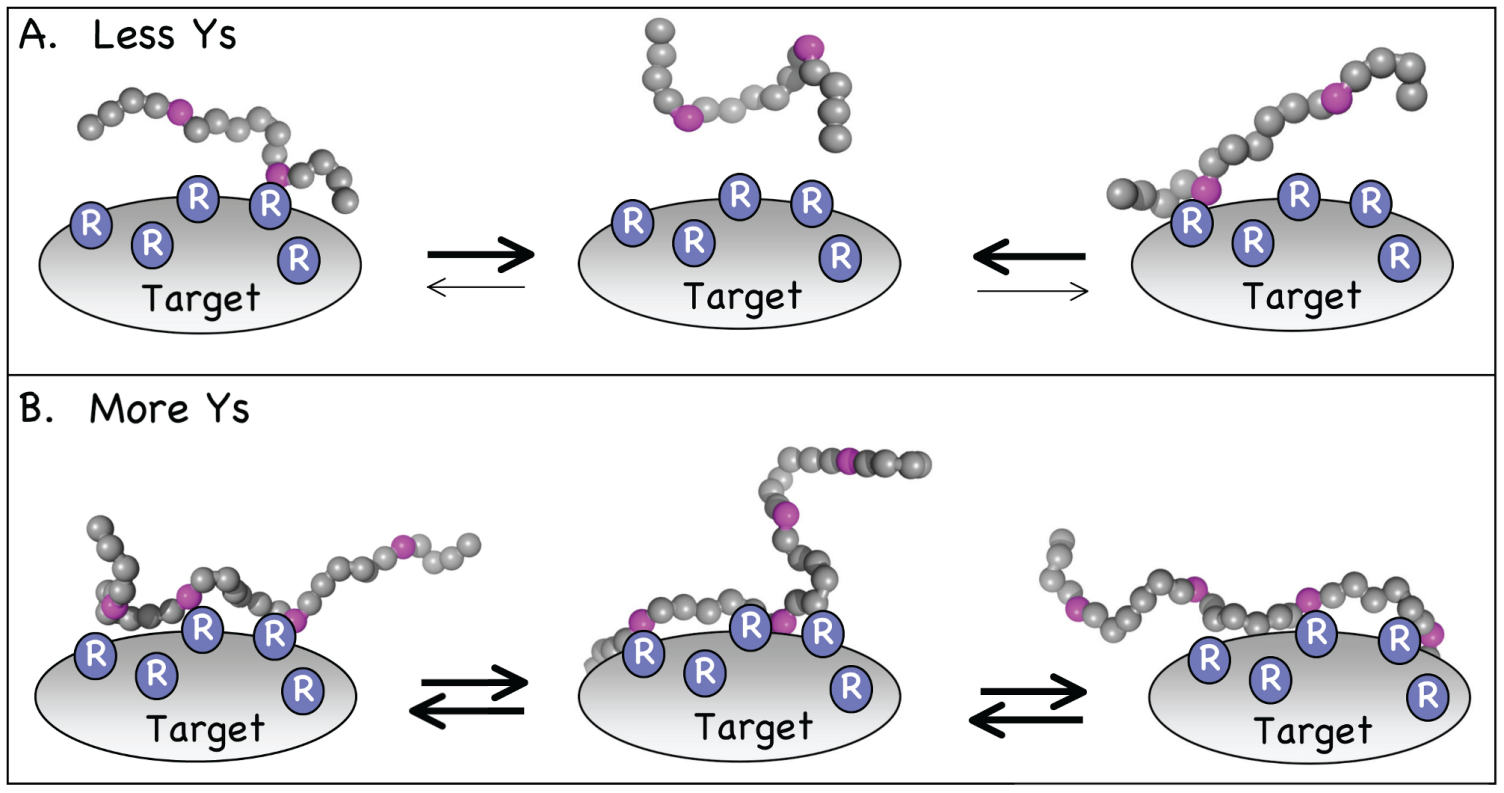
Model for molecular recognition by EAD. The EAD peptide is depicted here as a string of beads with aromatic (Y) residues in magenta and other residues in grey (see also Fig. S2). The target protein (Target) is generic and the number/distribution of surface positively charged (R) residues for real targets are unknown. Rs are chosen over Ks simply because Rs are more commonly paired with Ys in cation-π interactions. Binding is driven predominantly by cation-π interactions between Ys and Rs. A key postulate of the model is that the EAD remains disordered irrespective of binding and exists as a dynamic ensemble. Two general, high-probability states are depicted: (A) At low Y number the probability of EAD rebinding is low; dissociation is favored. (B) At higher Y number the probability of rebinding is sufficient to counteract dissociation and maintain binding.

The significance of protein disorder in the bound state or “fuzziness” has only recently emerged [6]. Theoretical modeling of IDPs [22], [49]–[67], especially for fuzzy complexes [22], is also in its infancy but provides powerful tools for understanding dynamic conformer ensembles. Our integrated functional and computational approach has culminated in a distinctive model for fuzzy interactions (Fig. 5) that may contain the core features of a more general mode of protein-protein interaction. The model involves a simple biophysical contact (cation-π), strong cooperativity stemming from both IDP and target polyvalency, and a highly flexible and dynamic IDP conformer population in the bound state. Stable binding requires a sufficient number of cation-π contacts but allows kinetic exchanges between myriad bound states. Notably the molecular recognition events studied here are particular to the diseased state of EFP-induced malignancies and are therefore of immediate biomedical interest.

### Robustness of the polycation-π model

Our hypothesis is intuitive given that cation-π interactions have wide and versatile biological roles, the interaction is strong [38] and EAD is highly polyvalent. The native intact EAD is also virtually devoid of cationic residues and thus especially amenable to trans cation-π interactions with target proteins. Here, our interrogation of the polycation-π model covered a wide range of EAD sequence properties (variations of Y number, cation-π strength, charge, Y density, and Y distribution) as well as simulation parameters (physically relevant variations of the cation-π, hydrophobic, and electrostatic interaction strengths; see Text S1). In all these tests, the polycation-π hypothesis provides a consistent biophysical account of the experiments. Other types of interactions are much less likely to contribute dominantly to EAD-target binding and our experiments address some of these. Of particular interest is the stoichiometric intramolecular blocking of Ys by Rs within EAD (Fig. 3C). This observation argues against alternative EAD-target aromatic interactions such as π-π stacking which are, in any event, probably of insufficient strength [68] in the absence of proximate metal ions [69] to account for the slope of Y number dependence of EAD activity (Fig. 1). One may also imagine a scenario in which EAD compaction is induced by Y-dependent hydrophobic interactions such that EAD-target contacts may involve poorly defined non-aromatic entities. But this possibility is strongly contraindicated by the high degree of EAD disorder [14] and also by our finding that EAD compaction by introduction of R residues (Text S1) actually decreases activity. Potential hydrogen bonding effects are not addressed in our model due to insufficient experimental data. Intuitively, hydrogen bonding involving prevalent EAD residues (Gln, Ser, and Thr) may well contribute to molecular recognition by EAD, although previous data [14], [25] together with the current study indicate that cation-π interactions are the essential driving force. More refined studies will be required to uncover secondary and more subtle contributions to EAD-target binding, including potential couplings between hydrogen bonding and cation-π interactions [70]. We also stress that our results do not preclude additional effects due to EAD posttranslational modifications, including tyrosine phosphorylation and O-GlcNAcylation [71], that might sometimes be manifest for particular EFPs and/or in specific physiological circumstances.

We have assumed a globular target because the biophysical aspects of the proposed model strongly predict that a large number of real globular proteins interact with EAD. Nonetheless, a disordered (IDP) target that enables favorable cation-π contacts with the EAD is also possible (Fig. S8) although so far the fuzzy complexes known to involve two IDPs are homodimers [72], [73]. We cannot infer how many cation-π contacts are required for EAD binding to real targets. It is also likely that particular interactions will deviate in some manner from our generic model. One can envision a variety of target determinants that might have an impact, including, for example, number and/or density of cations, acute geometric constraints imposed by residues flanking target cations, and the contribution of other aromatic side chain interactions such as hydrogen bonding.

### Comparison of polyelectrostatic and polycation-π interactions

Polyelectrostatic (Sic1/Cdc4) and polycation-π interactions share some similarities. Each may well reflect a general mode of interaction for polyvalent IDPs. In contrast to Sic1-Cdc4, however, the properties of the EAD studied herein are related to the diseased state [14] and our study points to several significant biophysical differences between EAD and Sic1/Cdc4. First, Sic1/Cdc4 binding involves a single Cdc4 site while EAD binding in our model invokes multiple simultaneous contacts. Second, Sic1/Cdc4 interaction is switch-like, reflecting the biological need for acute response to cell cycle kinase levels, whereas the EAD is constitutively polyvalent [14], [25]. Third, like most other polyvalent IDPs, Sic1 has short sequence-specific or linear motifs [74], [75], a single copy of which can mediate suboptimal or high-affinity Sic1/Cdc4 binding [10]. Such elements are almost certainly absent in EAD [14]. Fourth, the multiple cation-π contacts that underpin EAD binding in our model entail transient restrictions of EAD conformations (though they remain disordered), whereas a Sic1 bound to a single Cdc4 pocket at a given instant is not subject to such conformational restriction [22]–[24].

### Biological implications

The molecular recognition events studied here are related to pathological EAD function and, accordingly, are not obviously shaped by evolution [14]. Some aspects of EAD malfunction are an indirect consequence of loss of the EWS RNA-binding domain (RBD) or gain of a foreign DNA-binding domain in EFPs. In relation to our study, it is intriguing that the EWS RBD contains highly disordered regions with reiterated RGG that autorepress EAD [76], quite possibly via intramolecular masking [40]. The polycation-π perspective may offer a rationalization for this behavior. The simulated binding between a disordered EWS peptide containing multiple RGG boxes and the 10Yn EAD indeed reveals a strong interaction (Fig. S8). Intramolecular cation-π interactions between EAD and RGG have high potential to impact native EWS function by competing out aberrant interactions between EAD and the putative globular proteins relevant to EAD malfunction in oncogenesis.

In this regard, knowledge of EAD-target interface might provide therapeutic avenues [77] for Ewing's family tumors with poor prognosis. Several small molecule inhibitors of EWS/Fli1 have been identified. Interestingly, they all have aromatic character [78]–[80] or, in one case, a very basic short peptide sequence [81]. Whether any of them target the EAD portion of EWS/Fli is unknown. Due to their likely being effective cation-π competitors, it will be of great biomedical interest to explore this possibility.

How may polyaromatic molecular recognition by EAD relate to normal EWS protein function? This is a challenging question given the strong evolutionary conservation of EWS [82] that includes several EAD properties: a positionally conserved Gln two residues C-terminal to Y, Y phosphorylation sites [83], and SH2/SH3 interaction sites. However, none of these features are required, at least in some cases, for oncogenic EAD function [14]. Perhaps the mode of EAD action in EFP oncoproteins reflects a primordial polyaromatic function that was subsequently tailored by evolution to fulfill normal cellular roles. For example, Y phosphorylation can dramatically increase the aromatic-cation interactions required for peptide inhibitors of Src [84], indicating that phosphorylation of only a limited number of Ys in EAD could have profound effects on EAD-target interactions that are important for normal EWS.

To conclude, the proposed model for molecular recognition by EAD expands the seemingly endless modalities for IDP function and malfunction. The hitherto unrecognized polycation-π mode of IDP-target binding can be versatile. It offers a highly plausible biophysical basis for EAD and perhaps other scaffold/networking proteins to interact with many distinct target proteins [16]–[18]. The present methodology and results can also be extended to facilitate the exciting search for real EAD targets.

## Methods

### Experiment

*Plasmids*: pZΔE [25] and pZ7Luc [39] are previously described. All other plasmids expressing EAD variants were derived from the mammalian expression vector pSliencer 4.1-CMV neo (Applied Biosystems). *Proteins*: pZΔE expresses a protein lacking EAD sequences and containing only the ATF1 region and zta bZIP domain [25]; see Fig. S1. *Transactivation assays and Western blotting*: Transfections, trans-activation assays and quantitation of transactivation under linear assay conditions were performed as previously described [25]. Activity values were corrected for background activity determined by including the EAD-negative protein ZΔE in transfections. Details for plasmid and EAD construction and the assays are provided in Text S1.

### Simulation

The EAD is modeled as a C_α_ chain. Pairwise interactions between amino acid residues depend on whether they are aromatic, hydrophobic, charged, or polar (see Text S1 and Fig. S2E,F for definition). The generic EAD-binding target is a sphere of radius 16.0 Å with 32 positively and 32 negatively charges on its surface (Fig. S2A). The total energy of the model system 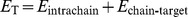 is the sum of the intramolecular energy 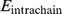 within the EAD and the intermolecular energy 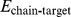 between the EAD and its target. The expressions for these energy functions, other modeling details, and control simulations are provided in Text S1.

## Supporting Information

Figure S1 **Proteins and EAD sequences used in the present study.** Transcriptional activator proteins (*Top*) contain the experimental sequences related to the N-terminal 66 residues of EAD1-66 (box with purple Ys), the region of ATF1 protein (ΔATF1) present in the EWS/ATF1 oncogene and the DNA-binding domain of zta protein (ztaDBD). In (A)–(C), amino acid residues are denoted by the standard one-letter code. Sequences for Figs. 1, 3, and 4 in the main text are listed, respectively, under (A), (B) and (C).
(JPG)

Figure S2
**The chain simulation model.** (A) The generic EAD binding target (partner) is a sphere of radius 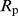 = 16 Å with essentially evenly distributed positive and negative charges (represented by blue and red beads respectively). (B) An EAD sequence is modeled as a 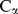 chain (beads on a string) that can engage in cation-π, electrostatic, hydrophobic, and excluded-volume interactions as specified in the main text and Text S1. In this figure and subsequent supporting figures, aromatic (Y in this drawing) and hydrophobic (hφ) residues are shown in magenta and orange, respectively, whereas positively and negatively charged residues are shown in blue and red respectively. All other residues are shown in grey. (C) The distribution of positively charged residues on the heterodimer of the Rpb4/Rpb7 subunits of human RNA polymerase II was used as a reference for the design of the charge density on the generic EAD binding target. The histogram here shows the shortest distance from each of the 32 positively charged amino acid residues (R or K) on Rpb4/Rpb7 (16 each along the Rpb4 and Rpb7 chains) from another positively charged residue, based on the X-ray crystal structure (PDB ID: 2C35) determined by Meka et al. (ref. [10] of Text S1). The distances are measured between the atoms that have the positive charges. The red dashed horizontal line marks the average shortest distance which is ≈9.4 Å. (D) EAD-target binding is defined in the model as having at least one EAD aromatic residue (magenta circle) within a capture radius 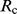 = 6 Å from a positive charge (blue circle) on the target. One such cation-π contact between an EAD sequence (brown string connecting magenta circles) and the target (large shaded circle with embedded blue circles) is shown in this schematic drawing. (E,F) Energetic components of the interaction potential, the horizontal variable *r* here corresponds to 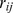 in Eq. (**S1**) or 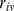 in Eq. (**S2**). (E) Model cation-π interaction potentials in the form of 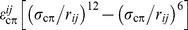 or 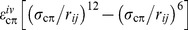 in Eqs. (**S1**) and (**S2**) respectively [i.e., equivalent to Fig. 1B in the main text minus the 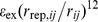 term]. The green and blue curves show the potentials for cation-W and cation-Y, respectively, as in Fig. 1B, whereas the red curve corresponds to the weakest among the model cation-F interactions considered in Fig. 1B. (F) Total interaction potential between hydrophobic residues and between charged residues in the simulation chain model, including their respective excluded-volume interactions. Solid curves show potential functions used for all simulation results presented in this work except specifically noted otherwise. Dashed curves show alternative potential functions that we have used for the control simulations reported in Text S1. The potential functions used for hydrophobic interaction are shown in magenta. The solid curve is for hydrophobic interaction strength 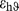 = −3.0 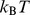 [Eq. (**S1**)] whereas the dashed curve is for 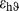 = −7.0 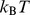. The potential functions for electrostatic interactions between like charges and between opposite charges are shown, respectively, in red and blue. The solid curves are for 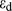 = 40 whereas the dashed curves are for 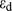 = 20.(PDF)

Figure S3
**Evidence for the polycation-π hypothesis from a re-analysis of early experiments on 33-residue EAD sequences.** Sequences and experimental data were taken from ref. [1] of Text S1. Simulations were conducted using the same chain model as described in Text S1 and the main text in a (600 Å)^3^ simulation box. (A) The sequences are defined in the above reference. The experimental relative activities and the simulated relative binding probabilities are represented by the black and grey bars respectively. (B) The sequences in (A) are grouped according to their Y number 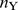. Plotted are the simulated binding probability (solid squares) and the relative experimental activity (open circles) averaged over sequences belonging to each given 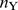. For the simulation results, the averages are over all possible permutations of Y positions for a given 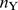, including those not studied by experiments. Note that both Y number and Y density are varied among this set of sequences (unlike the set in Fig. 1 that varies only the Y number while keeping Y density constant). Error bars show variation among sequences with the same 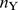. Lines joining the solid squares are merely a guide for the eye.(PDF)

Figure S4
**Simulated binding probabilities of monomer and dimer EAD sequences follow similar trends.** Similar dependences on 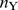 are observed for cis-duplication of small EAD elements in a single dimer. The monomer sequences used in the present simulations are the same 33-residue sequences based on the construction by Feng and Lee (ref. [1] of Text S1) studied in Fig. S3. As for the simulations in Fig. S3, all possible permutations of Y positions are considered. Each dimer was constructed by joining the C-terminus of a given monomer sequence to the C-terminus of another copy of the same monomer sequence by a linker chain. The linker consists of six residues that are neither charged nor hydrophobic; all reference bond angles within the linker are equal to 165° with a stiff bond-angle force constant equal to 10.0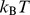. Thus, in this figure, a dimer sequence with Y number 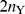
is equivalent to two identical monomer sequences with Y number 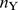
connected by such a linker. (A) A snapshot of an 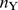 = 5 monomer bound to the target. (B) A snapshot of the corresponding 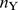 = 10 dimer bound to the target. The EAD chains are depicted in a tube representation with the color code for different residue types specified in Fig. S2B. (C) Free energies of binding were computed under the same conditions as those used for Fig. S3. 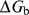 values averaging over sequences with the same 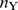 are plotted.(PDF)

Figure S5
**Components of the analytical model.** (A) Schematic of cation-π contacts along the IDP. Here we only consider IDP chains with evenly spaced aromatics that are *k* residues apart; thus the contour length between two cation-contacting aromatics is always in the form of 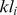 where 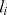 is a positive integer. Three example contact patterns are shown, wherein the aromatics and cations are depicted as magenta and blue circles respectively. (B) Distribution of cation-cation distance 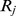 on the target. Each 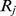 value is the distance in Å from a given cation to a different cation, measured on the spherical surface of the model target (left drawing). The distribution 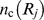 is shown (histograms) for three different targets of the same size but different cation densities. As for the target with 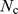 = 32 cations in most of our simulations, the cations are essentially evenly distributed on the surface for the 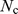 = 8 and 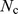 = 96 targets. The approximately even distribution of charges on the target sphere was achieved by a numerical algorithm (see Text S1). As can be seen from the histograms, only a few of the 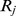 values are exactly identical. (C) An example conformation configured in the simple cubic lattice with one end of the chain touching a plane. The number of such conformations is referred to as 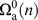 in this work. (D) An example simple cubic lattice conformation with two of its mid-chain sites in contact with a plane. We denote the number of such conformations as 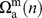. (E) Change in conformational entropy (in units of the Boltzmann constant 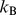) upon bringing a free lattice conformation to form a contact at a chain end (squares) or at mid-chain (circles) with an infinite impenetrable plane that imposes excluded volume on the other side of the plane (the space underneath the plane is not accessible to the chain). The data points (squares or circles) were computed using exact enumeration data in Table S1. The curves through the data points were generated by fitting the assumed relation 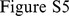. The fitting parameters here are *A* = 0.5365, *B* = 0.53139, *ω* = 0.02786, and *σ* = 0.33604 for 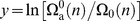; and *A* = 0.40915, *B* = 1.12627, *ω* = 0.05373, and *σ* = 0.39353 for 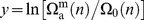.(PDF)

Figure S6
**Conformational entropy loss upon loop formation.** The quantity 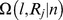 is the number of simple cubic lattice conformations of length *n* (*n* is the total number of beads along the chain) that have one chain end (bead number 1) touching an excluded-volume plane at a given point (as in Fig. S5C) and, at the same time, bead number *l*+1 also making a contact with a given point on the plane at a distance 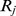 from where bead number 1 touches the plane, thus forming a loop of length *l* that spans a distance 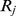 on the plane (top left drawing). Note that conformations that form other chain-plane contact(s) in addition to these two are included in the 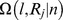 count. As discussed in the main text and in Text S1, the vertical variable 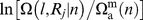 for the plots in this figure corresponds approximately to the conformational entropy change, in units of 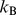, upon making an additional chain-plane contact to form a loop of length *l* along a chain that has already made at least one contact with the plane. Each of the plotting panels provides the conformational entropy change upon forming a loop of a given length *l* as a function of 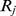. Both *l* and 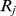 are shown in units of the lattice bond length (nearest distance between two beads on the simple cubic lattice). Data points (open circles) in the plotting panels were computed by exact enumeration of lattice conformations with chain lengths from *n* = 4 through *n* = 17 (see Text S1 and Tables S2 and S3). Multiple data points for the same 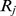 value represent results from different *n* values. The continuous curves are quadratic fits in the form of 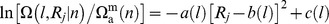. The *l*-dependent fitting parameters 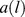, 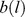, and 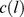 are provided in Fig. S7. In view of the clustering of data points from different *n* values, we have made an approximation in the analytical model that 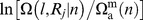 is independent of *n*.(PDF)

Figure S7
**Applying the lattice conformational entropy estimates to the analytical model.** (A–C) The fitting parameters 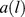, 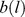, and 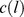 for the conformational entropy changes shown in Fig. S6 are provided as data points in (A), (B), and (C), respectively. The continuous fitting curves are given by (A) 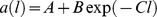, where *A* = 0.13748, *B* = 7.04181, and *C* = 0.52115; (B) 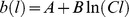, where *A* = 0.97499, *B* = 0.93564, and *C* = 0.97495; and (C) 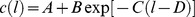, where *A* = −5.19530, *B* = 2.98286, *C* = 0.31975, and *D* = 2.79004. These expressions were used to estimate 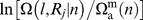 for *l*>16 by extrapolation. (D) The extrapolated 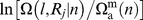 function (black curve) is compared against the corresponding random-flight expression 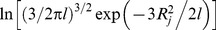 (red dashed curve) for *l* = 60. (E) Two methods for estimating the entropic cost of loop formation in the analytical model are compared. Plotted are the binding free energies of the model EAD chains in Fig. 1 for 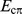 = −3.5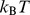. The black data points (circles) were computed by using entropy estimates from exact enumeration for *l*≤16 and extrapolated estimates for *l*>16, whereas the red data points (triangles) were obtained by using entropy estimates from exact enumeration for *l*≤16 but random-flight estimates for *l*>16. The plot here shows that the predicted 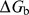 values based on the two different loop entropy estimates are very similar.(PDF)

Figure S8
**Exploring other EAD-target binding scenarios.** The EAD sequences are the same as those in Fig. 1. (A) Simulated EAD binding probability 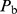 with a hypothetical target in which the surface charges are not evenly distributed but confined to a patch. Two such hypothetical patch partners were considered, both with 12 cations localized on a patch with the same local cation density as the generic target with 32 cations (Fig. S2A) that we have used for most of the simulations. One of the targets (referred to as the positive patch target) contains 12 cations and no anions on the patch whereas the other (referred to as the neutral patch target) contains 12 cations and 12 anions. Plotted here are the simulated binding probabilities for the positive (squares) and neutral (circles) patch targets in either a simulation box of size of (300 Å)^3^ (black symbols) or (600 Å)^3^ (blue symbols). (B) A snapshot of an 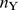 = 10 EAD sequence (tube representation) bound to the neutral patch target. (C) Simulated EAD binding probability 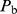 with hypothetical disordered (IDP) partners. The EAD sequences and simulation conditions are the same as those in Fig. 1B,C, using a simulation box of size (600 Å)^3^. During the binding simulations, both the EAD and the hypothetical IDP target were allowed to sample all accessible conformations while the center of mass of the IDP target was kept at a fixed position in the center of the simulation box. We considered a class of such targets, each of which is a chain consisting of 64 alternating cations and anions (32 cations and 32 anions). The adjacent cation and anion are connected by a 5 Å virtual bond with a stiff bond-angle force constant equal to 10.0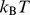. Shown here are binding probabilities for four different such IDP targets with equilibrium bond angles that equal, respectively, to 105° (crosses), 120° (diamonds), 135° (squares) and 150° (circles). A general trend of increasing binding with increasing 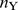 is observed for all four hypothetical IDP targets. Not surprisingly, the quantitative details of this trend are sensitive to the persistence length of the IDP target. Binding increases with the flexibility of the IDP target. Also included for comparison (blue triangles) are the simulated probabilities of EAD binding with the RGG3 sequence in the Ewing's sarcoma RNA-binding domain GGDRGRGGPGGMRGGRGGLMDRGGPGGMFRGGRGGDRGGFRGGRGMDRGGFGGGRRGGPGG (refs. [27], [28] in Text S1). Here the RGG3 sequence was modeled as a 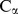 chain using the same modeling scheme as that for the EAD sequences. (D) A snapshot of an 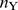 = 10 EAD sequence (tube representation) bound to a hypothetical IDP target (red and blue beads) with 150° bond angles.(PDF)

Table S1
**Numbers of conformations, or self-avoiding flights, on the simple cubic lattice.** Conformational counts as functions of chain length (number of beads) *n* are obtained by exact enumeration. A chain with *n* beads has *n*−1 bonds. Here, 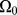 is the number of unconstrained conformations; 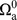 is the number of conformations that have one chain end anchored onto an impenetrable plane (Fig. S5C); and 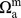 is the number of conformations that have the mid-chain bead [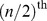 bead if *n* is even, 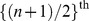 bead if *n* is odd] making a contact with an impenetrable plane (Fig. S5D).(PDF)

Table S2
**Loop probabilities determined by exact lattice conformational enumeration.** Tabulated here are examples (not a complete list) of conformational counts 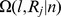 used in Fig. S6. Here one chain end is always in contact with the origin (0,0) of a two-dimensional coordinate system for the impenetrable plane. In this table, the positions on the impenetrable plane where another contact with the chain existed are indicated by the (*x*,*y*) coordinates. In the present treatment of our analytical model, 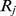 values from all combinations of *x*,*y* (where *x*<*y*) that have nonzero 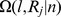 counts for *n*≤17 were used to estimate the conformational entropic cost of loop formation (Figs. S6 and S7).(PDF)

Table S3
**Exact lattice enumeration data for loop formation probability.** Tabulated here as examples are the exact 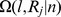 counts for *l* = 16 and *n* = 17. The horizontal and vertical labels correspond, respectively, to the *x* and *y* coordinates of the positions on the impenetrable plane. One end of the chain (first bead) is always anchored at the origin (0,0). In this table, the entry at a given position (*x*,*y*) is the number of conformations that have the chain's last (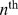) bead contacting the given position and thus making a loop with 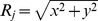. Data are shown only for *x*≤*y* because of the obvious rotational symmetry.(PDF)

Text S1 **Experimental and Computational Details and Rationale.**
(PDF)
