# Supplementary figures and images for: Polycation-π Interactions Are a Driving Force for Molecular Recognition by an Intrinsically Disordered Oncoprotein Family
Source: PLoS Comput Biol. 2013 Sep 26;9(9):e1003239. doi: 10.1371/journal.pcbi.1003239 (PMC3784488; doi:10.1371/journal.pcbi.1003239)

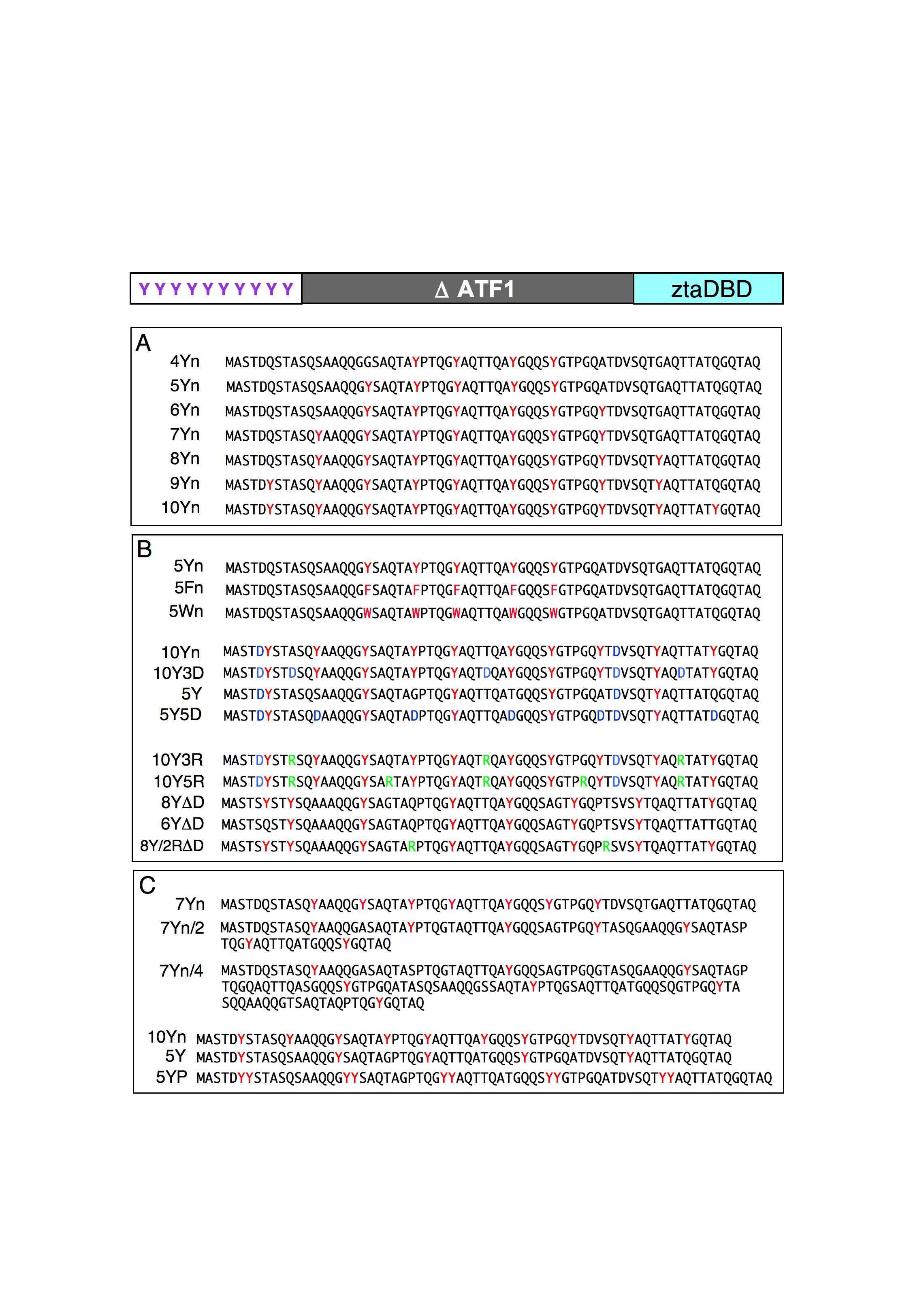

Supplement: Figure S1 — Proteins and EAD sequences used in the present study. Transcriptional activator proteins (Top) contain the experimental sequences related to the N-terminal 66 residues of EAD1-66 (box with purple Ys), the region of ATF1 protein (ΔATF1) present in the EWS/ATF1 oncogene and the DNA-binding domain of zta protein (ztaDBD). In (A)–(C), amino acid residues are denoted by the standard one-letter code. Sequences for Figs. 1, 3, and 4 in the main text are listed, respectively, under (A), (B) and (C). (JPG) [file pcbi.1003239.s001.jpg]

(A)

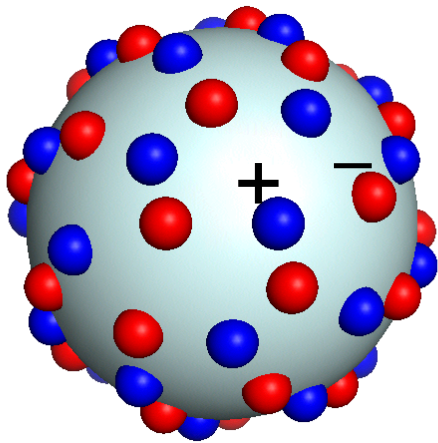

(B)

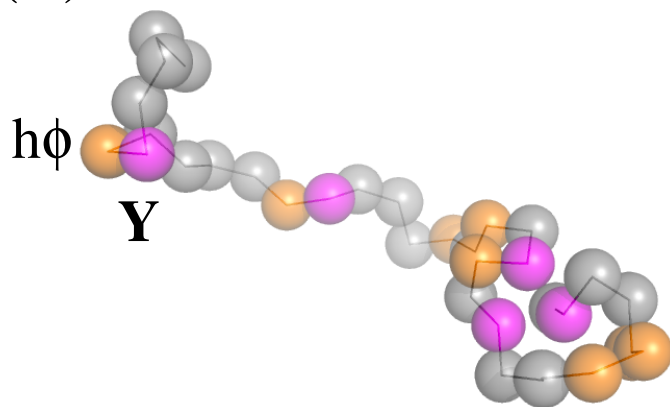

(C)

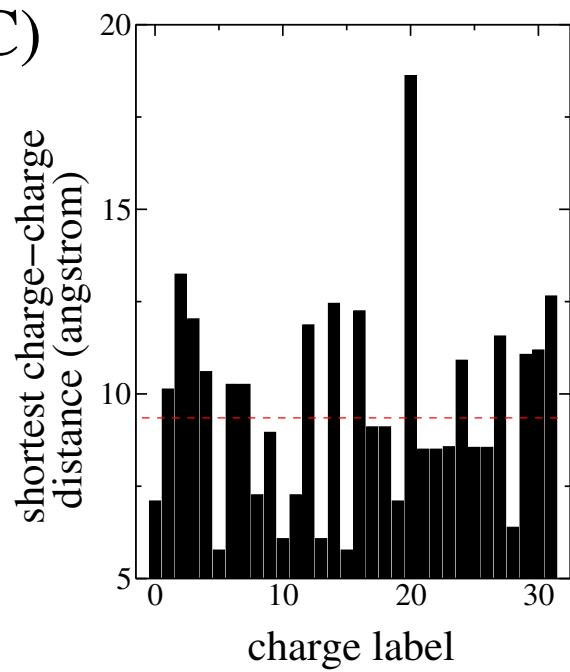

(D)

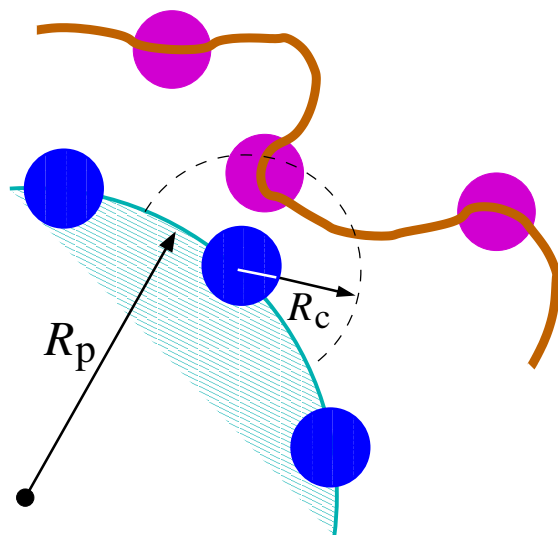

(E)

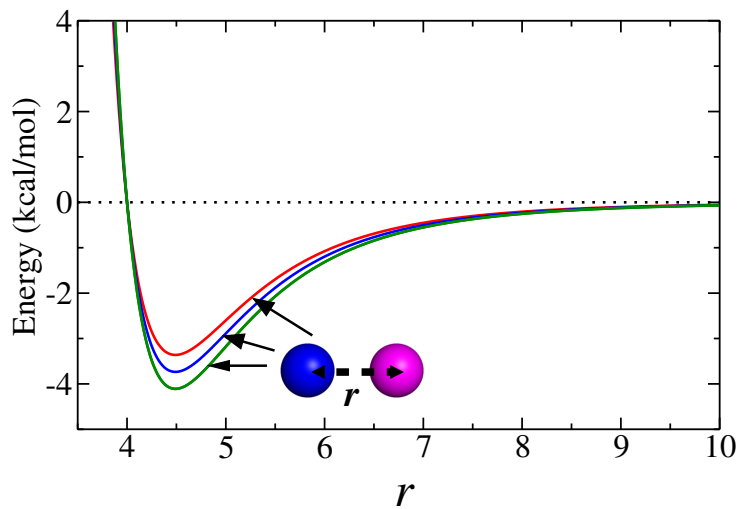

(F)

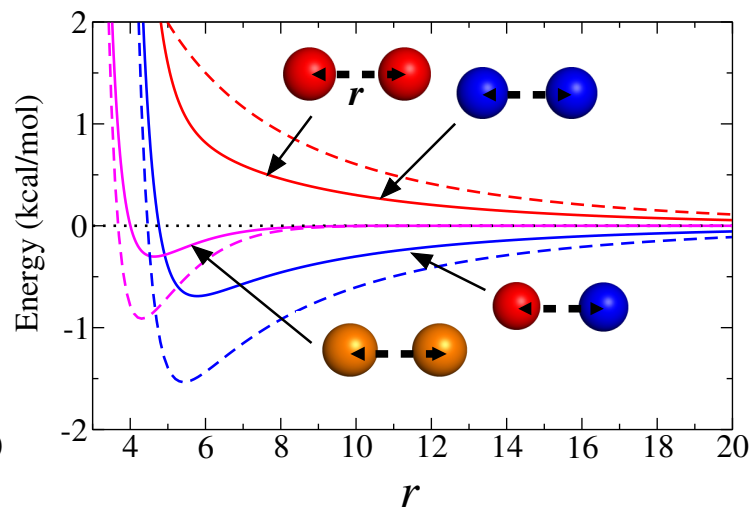

Supplement: Figure S2 — The chain simulation model. (A) The generic EAD binding target (partner) is a sphere of radius = 16 Å with essentially evenly distributed positive and negative charges (represented by blue and red beads respectively). (B) An EAD sequence is modeled as a chain (beads on a string) that can engage in cation-π, electrostatic, hydrophobic, and excluded-volume interactions as specified in the main text and Text S1. In this figure and subsequent supporting figures, aromatic (Y in this drawing) and hydrophobic (hφ) residues are shown in magenta and orange, respectively, whereas positively and negatively charged residues are shown in blue and red respectively. All other residues are shown in grey. (C) The distribution of positively charged residues on the heterodimer of the Rpb4/Rpb7 subunits of human RNA polymerase II was used as a reference for the design of the charge density on the generic EAD binding target. The histogram here shows the shortest distance from each of the 32 positively charged amino acid residues (R or K) on Rpb4/Rpb7 (16 each along the Rpb4 and Rpb7 chains) from another positively charged residue, based on the X-ray crystal structure (PDB ID: 2C35) determined by Meka et al. (ref. [10] of Text S1). The distances are measured between the atoms that have the positive charges. The red dashed horizontal line marks the average shortest distance which is ≈9.4 Å. (D) EAD-target binding is defined in the model as having at least one EAD aromatic residue (magenta circle) within a capture radius = 6 Å from a positive charge (blue circle) on the target. One such cation-π contact between an EAD sequence (brown string connecting magenta circles) and the target (large shaded circle with embedded blue circles) is shown in this schematic drawing. (E,F) Energetic components of the interaction potential, the horizontal variable r here corresponds to in Eq. (S1) or in Eq. (S2). (E) Model cation-π interaction potentials in the form of or in Eqs. (S1) and (S2) respectively [ [file pcbi.1003239.s002.pdf]

(A) relative activity /  
simulated binding

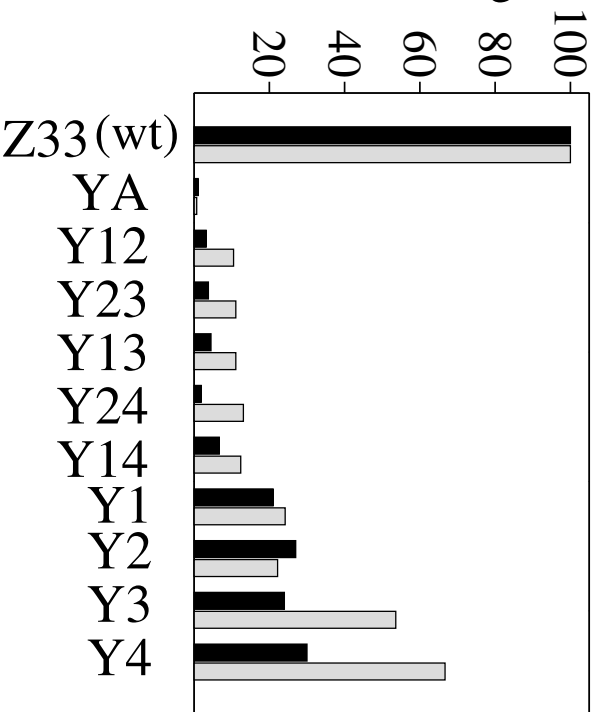

(B)

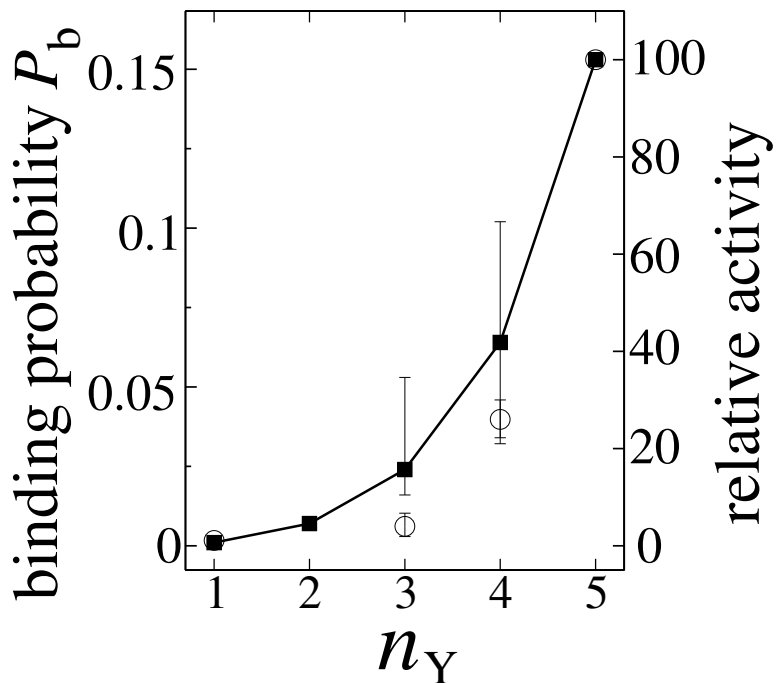

Supplement: Figure S3 — Evidence for the polycation-π hypothesis from a re-analysis of early experiments on 33-residue EAD sequences. Sequences and experimental data were taken from ref. [1] of Text S1. Simulations were conducted using the same chain model as described in Text S1 and the main text in a (600 Å)3 simulation box. (A) The sequences are defined in the above reference. The experimental relative activities and the simulated relative binding probabilities are represented by the black and grey bars respectively. (B) The sequences in (A) are grouped according to their Y number . Plotted are the simulated binding probability (solid squares) and the relative experimental activity (open circles) averaged over sequences belonging to each given . For the simulation results, the averages are over all possible permutations of Y positions for a given , including those not studied by experiments. Note that both Y number and Y density are varied among this set of sequences (unlike the set in Fig. 1 that varies only the Y number while keeping Y density constant). Error bars show variation among sequences with the same . Lines joining the solid squares are merely a guide for the eye. (PDF) [file pcbi.1003239.s003.pdf]

(A)

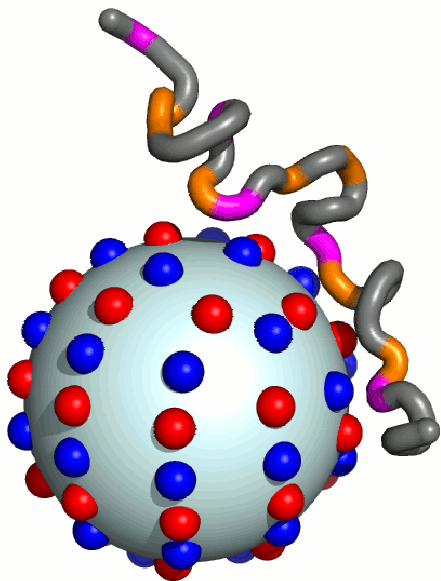

(B)

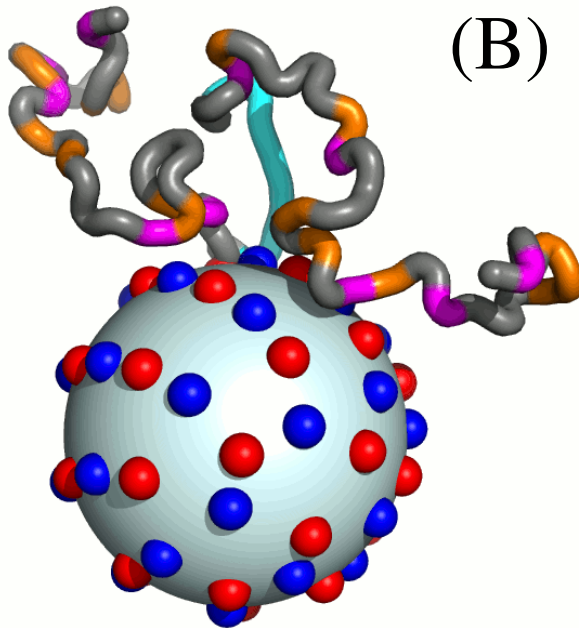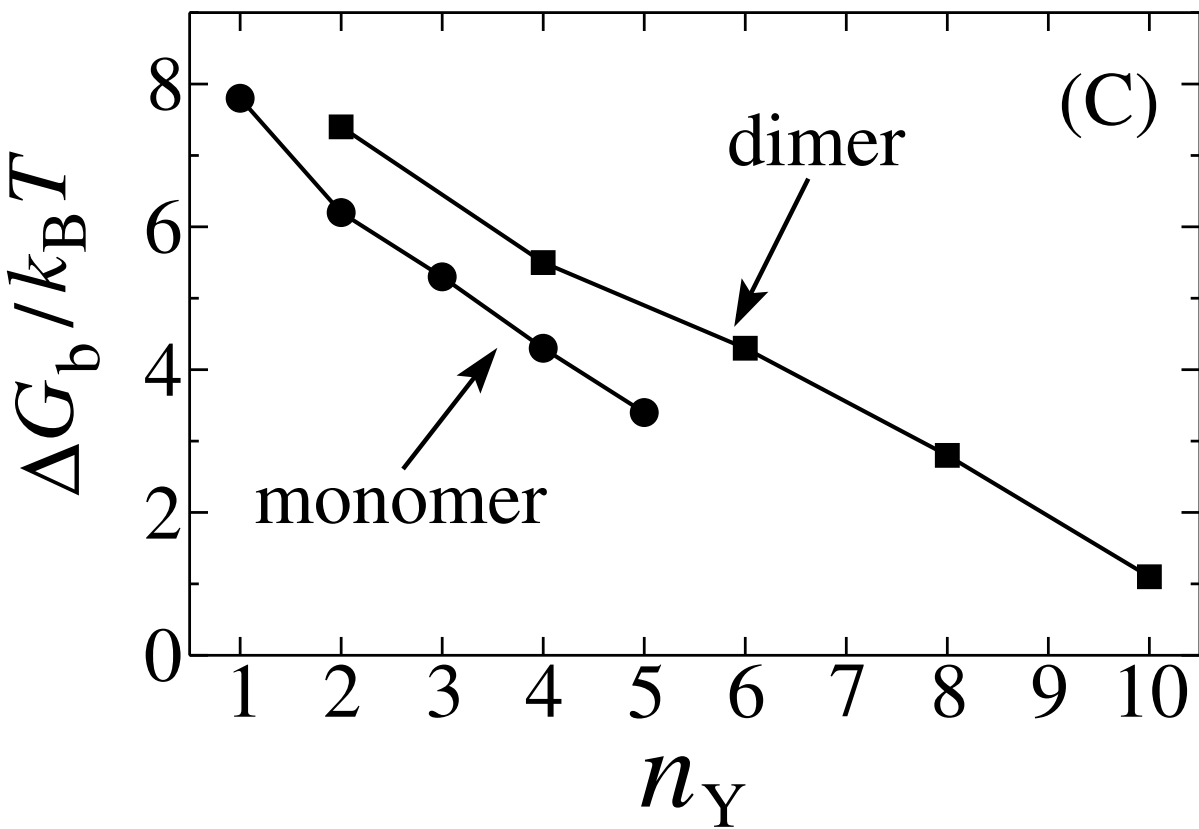

Supplement: Figure S4 — Simulated binding probabilities of monomer and dimer EAD sequences follow similar trends. Similar dependences on are observed for cis-duplication of small EAD elements in a single dimer. The monomer sequences used in the present simulations are the same 33-residue sequences based on the construction by Feng and Lee (ref. [1] of Text S1) studied in Fig. S3. As for the simulations in Fig. S3, all possible permutations of Y positions are considered. Each dimer was constructed by joining the C-terminus of a given monomer sequence to the C-terminus of another copy of the same monomer sequence by a linker chain. The linker consists of six residues that are neither charged nor hydrophobic; all reference bond angles within the linker are equal to 165° with a stiff bond-angle force constant equal to 10.0. Thus, in this figure, a dimer sequence with Y number is equivalent to two identical monomer sequences with Y number connected by such a linker. (A) A snapshot of an = 5 monomer bound to the target. (B) A snapshot of the corresponding = 10 dimer bound to the target. The EAD chains are depicted in a tube representation with the color code for different residue types specified in Fig. S2B. (C) Free energies of binding were computed under the same conditions as those used for Fig. S3. values averaging over sequences with the same are plotted. (PDF) [file pcbi.1003239.s004.pdf]

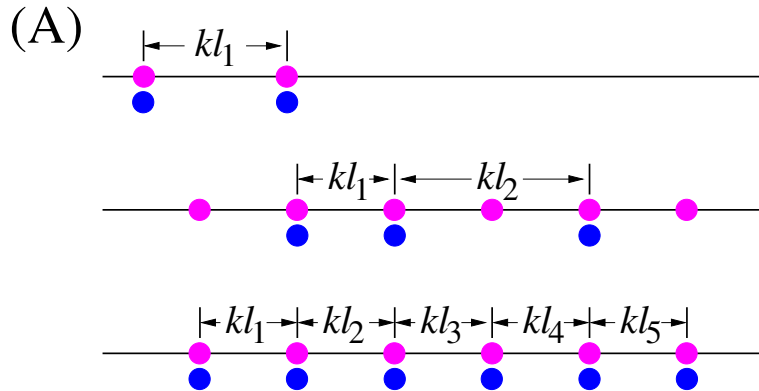

(B)

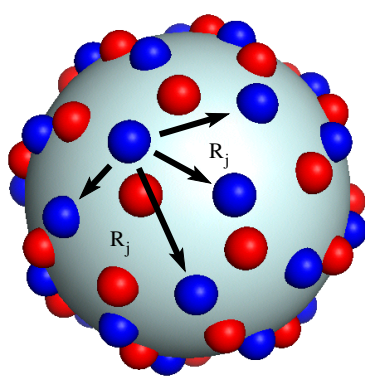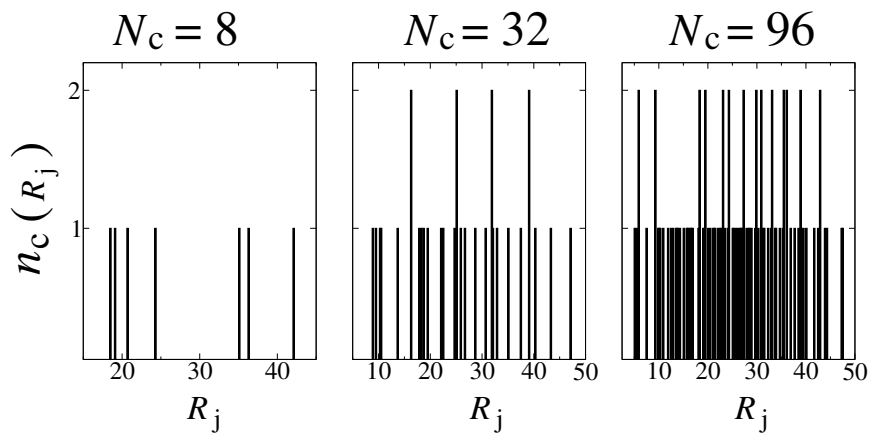

(C)

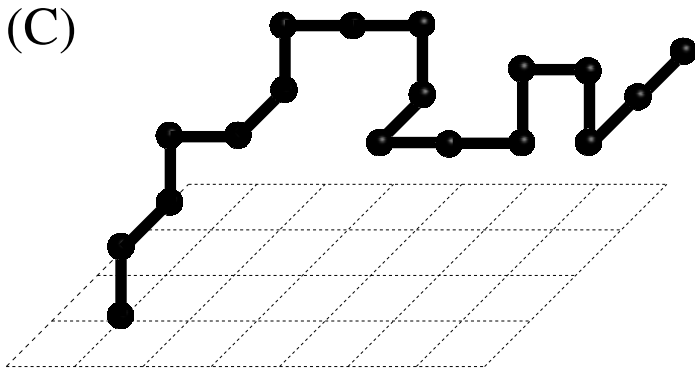

(D)

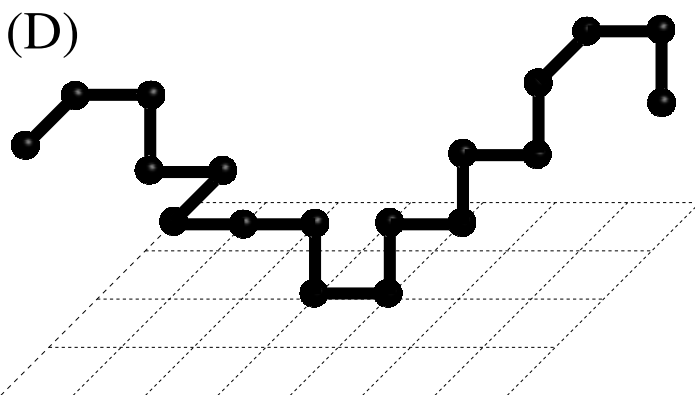

(E)

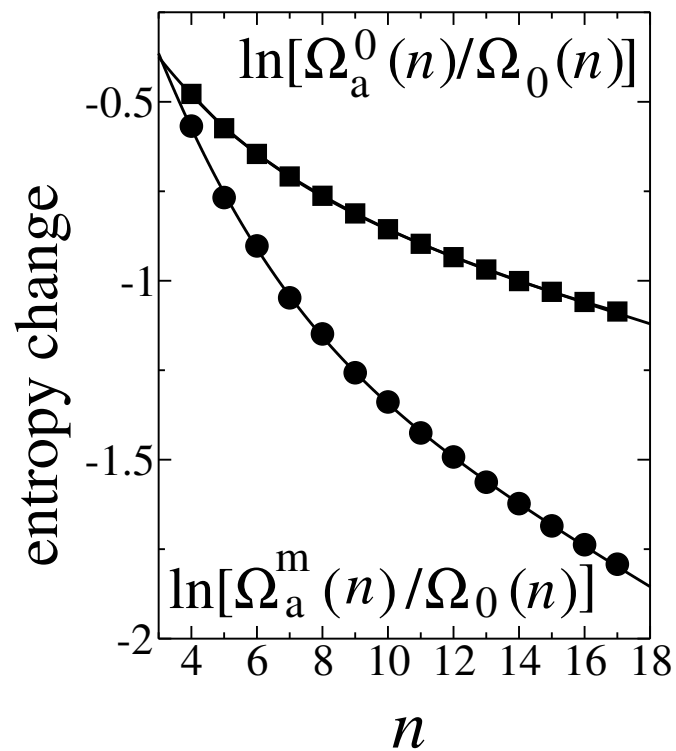

Supplement: Figure S5 — Components of the analytical model. (A) Schematic of cation-π contacts along the IDP. Here we only consider IDP chains with evenly spaced aromatics that are k residues apart; thus the contour length between two cation-contacting aromatics is always in the form of where is a positive integer. Three example contact patterns are shown, wherein the aromatics and cations are depicted as magenta and blue circles respectively. (B) Distribution of cation-cation distance on the target. Each value is the distance in Å from a given cation to a different cation, measured on the spherical surface of the model target (left drawing). The distribution is shown (histograms) for three different targets of the same size but different cation densities. As for the target with = 32 cations in most of our simulations, the cations are essentially evenly distributed on the surface for the = 8 and = 96 targets. The approximately even distribution of charges on the target sphere was achieved by a numerical algorithm (see Text S1). As can be seen from the histograms, only a few of the values are exactly identical. (C) An example conformation configured in the simple cubic lattice with one end of the chain touching a plane. The number of such conformations is referred to as in this work. (D) An example simple cubic lattice conformation with two of its mid-chain sites in contact with a plane. We denote the number of such conformations as . (E) Change in conformational entropy (in units of the Boltzmann constant ) upon bringing a free lattice conformation to form a contact at a chain end (squares) or at mid-chain (circles) with an infinite impenetrable plane that imposes excluded volume on the other side of the plane (the space underneath the plane is not accessible to the chain). The data points (squares or circles) were computed using exact enumeration data in Table S1. The curves through the data points were generated by fitting the assumed relation . The fitting parameters here are A = 0.5365 [file pcbi.1003239.s005.pdf]

$\ln[\Omega(l, R_j | n) / \Omega_a^m(n)]$

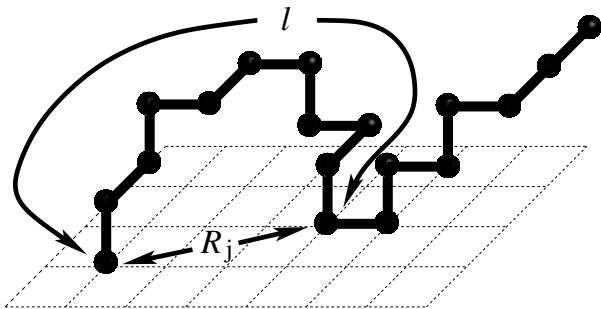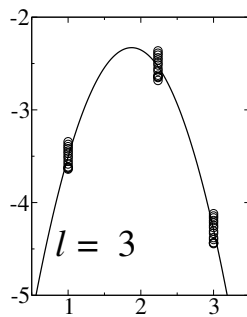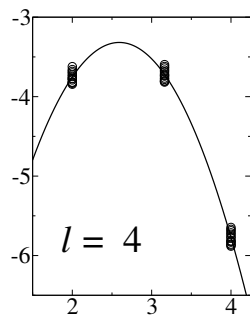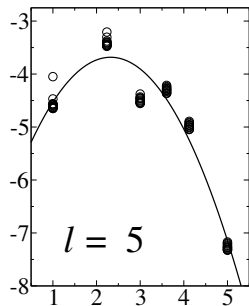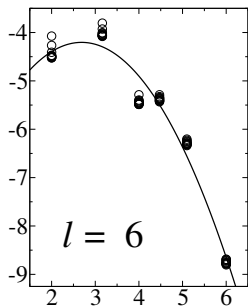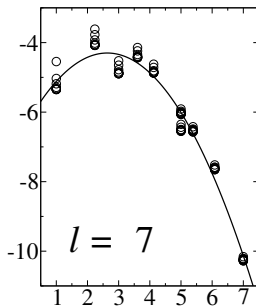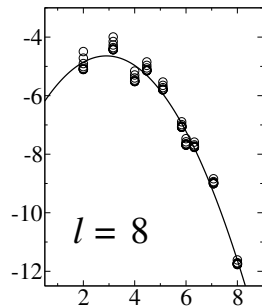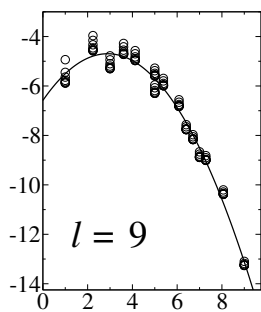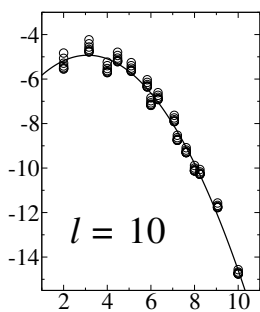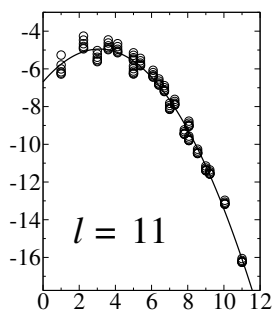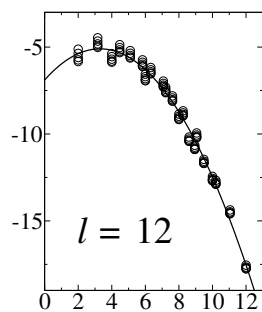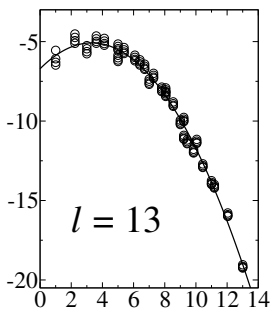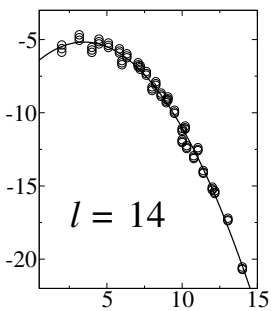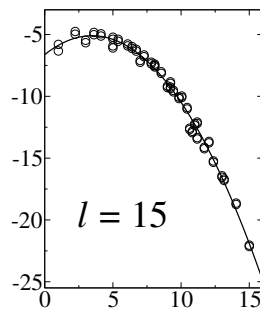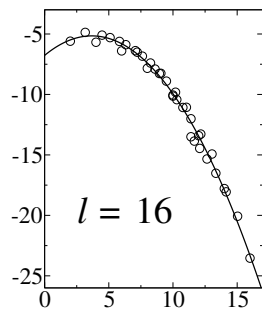

$R_j$

Supplement: Figure S6 — Conformational entropy loss upon loop formation. The quantity is the number of simple cubic lattice conformations of length n (n is the total number of beads along the chain) that have one chain end (bead number 1) touching an excluded-volume plane at a given point (as in Fig. S5C) and, at the same time, bead number l+1 also making a contact with a given point on the plane at a distance from where bead number 1 touches the plane, thus forming a loop of length l that spans a distance on the plane (top left drawing). Note that conformations that form other chain-plane contact(s) in addition to these two are included in the count. As discussed in the main text and in Text S1, the vertical variable for the plots in this figure corresponds approximately to the conformational entropy change, in units of , upon making an additional chain-plane contact to form a loop of length l along a chain that has already made at least one contact with the plane. Each of the plotting panels provides the conformational entropy change upon forming a loop of a given length l as a function of . Both l and are shown in units of the lattice bond length (nearest distance between two beads on the simple cubic lattice). Data points (open circles) in the plotting panels were computed by exact enumeration of lattice conformations with chain lengths from n = 4 through n = 17 (see Text S1 and Tables S2 and S3). Multiple data points for the same value represent results from different n values. The continuous curves are quadratic fits in the form of . The l-dependent fitting parameters , , and are provided in Fig. S7. In view of the clustering of data points from different n values, we have made an approximation in the analytical model that is independent of n. (PDF) [file pcbi.1003239.s006.pdf]

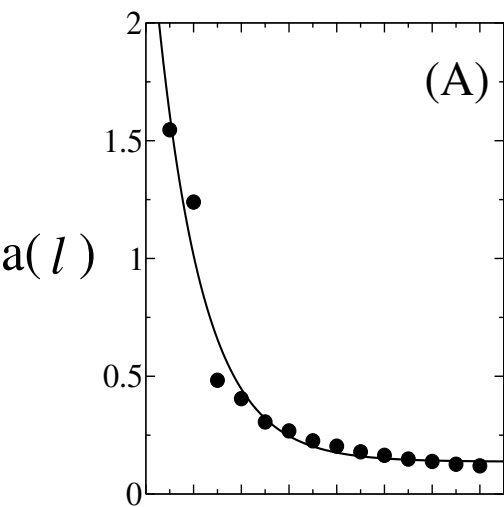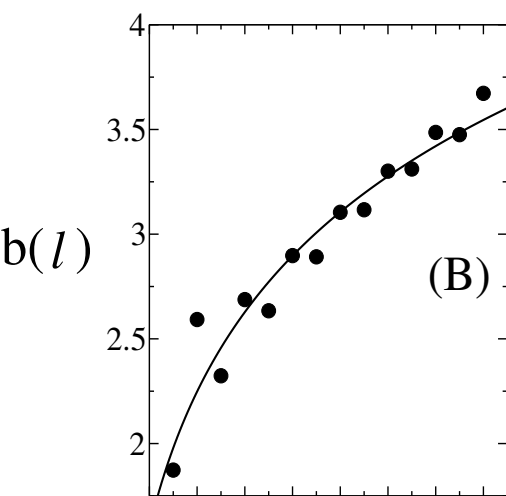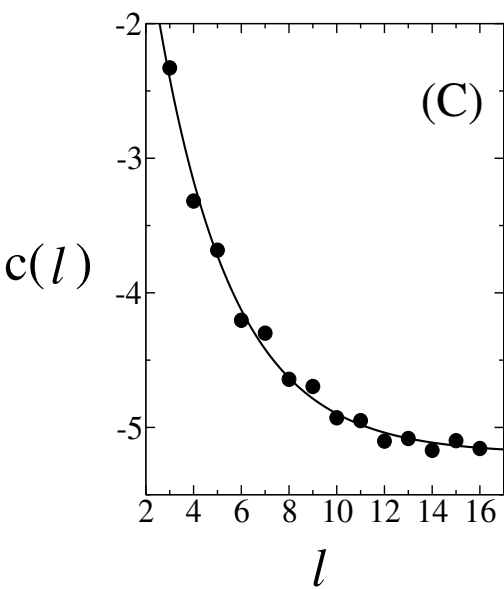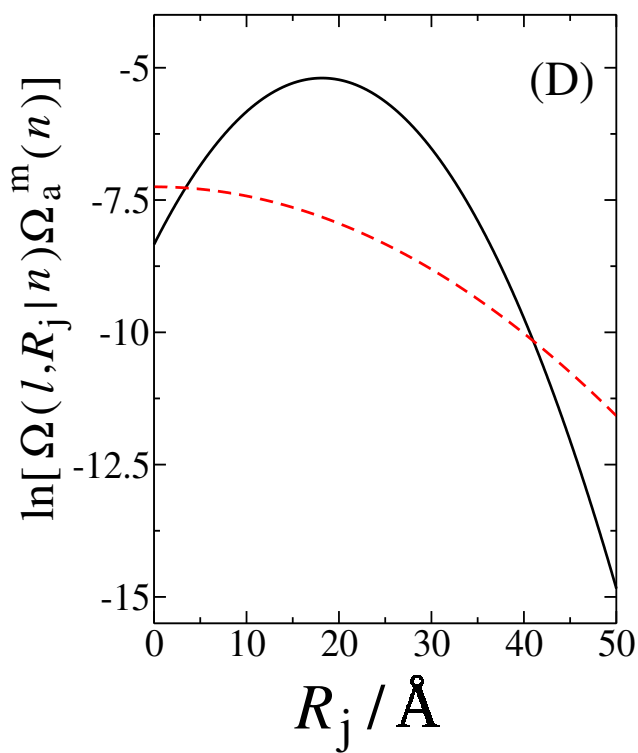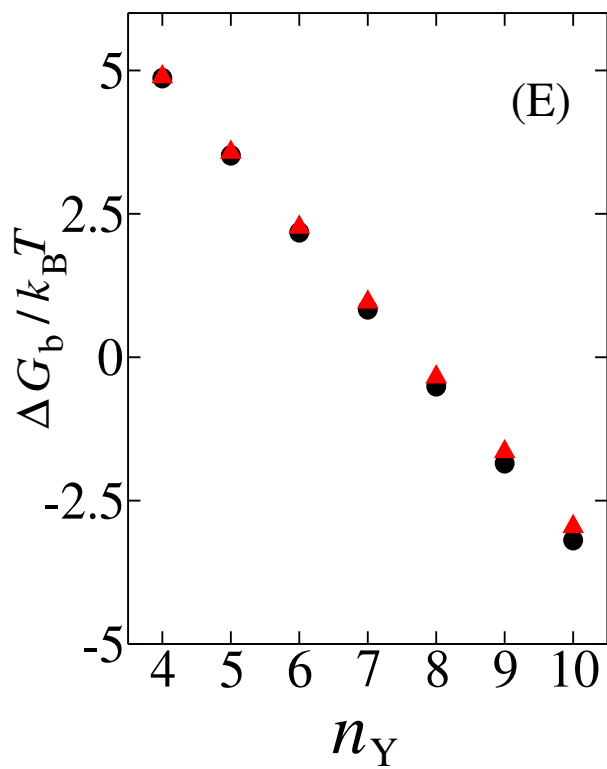

Supplement: Figure S7 — Applying the lattice conformational entropy estimates to the analytical model. (A–C) The fitting parameters , , and for the conformational entropy changes shown in Fig. S6 are provided as data points in (A), (B), and (C), respectively. The continuous fitting curves are given by (A) , where A = 0.13748, B = 7.04181, and C = 0.52115; (B) , where A = 0.97499, B = 0.93564, and C = 0.97495; and (C) , where A = −5.19530, B = 2.98286, C = 0.31975, and D = 2.79004. These expressions were used to estimate for l>16 by extrapolation. (D) The extrapolated function (black curve) is compared against the corresponding random-flight expression (red dashed curve) for l = 60. (E) Two methods for estimating the entropic cost of loop formation in the analytical model are compared. Plotted are the binding free energies of the model EAD chains in Fig. 1 for = −3.5. The black data points (circles) were computed by using entropy estimates from exact enumeration for l≤16 and extrapolated estimates for l>16, whereas the red data points (triangles) were obtained by using entropy estimates from exact enumeration for l≤16 but random-flight estimates for l>16. The plot here shows that the predicted values based on the two different loop entropy estimates are very similar. (PDF) [file pcbi.1003239.s007.pdf]

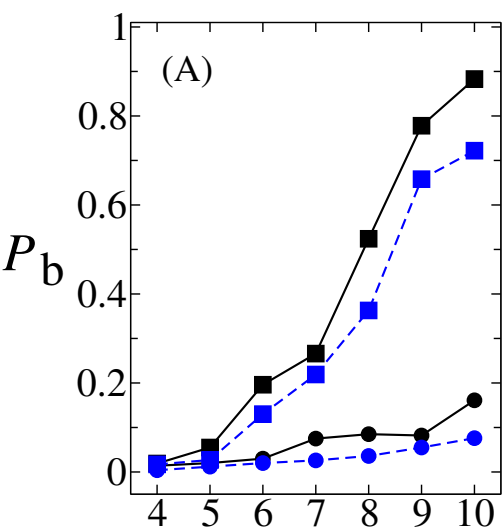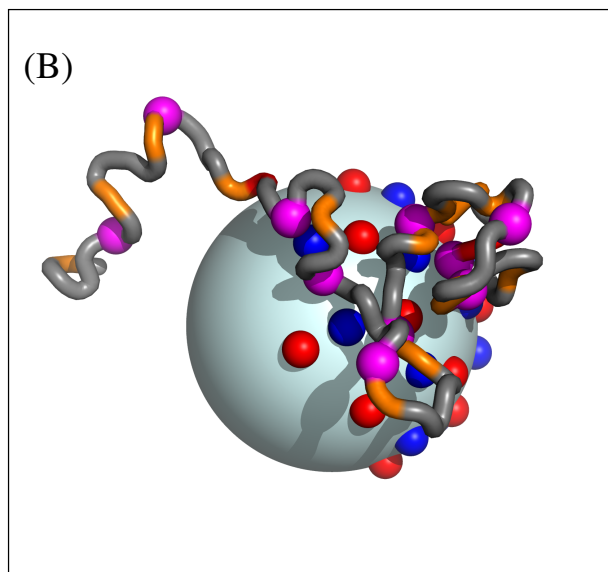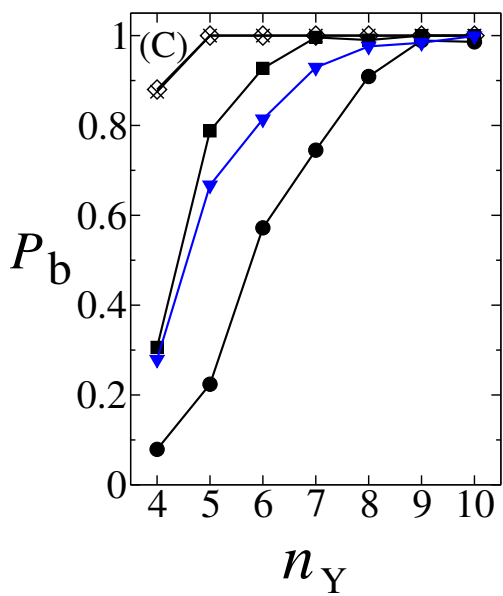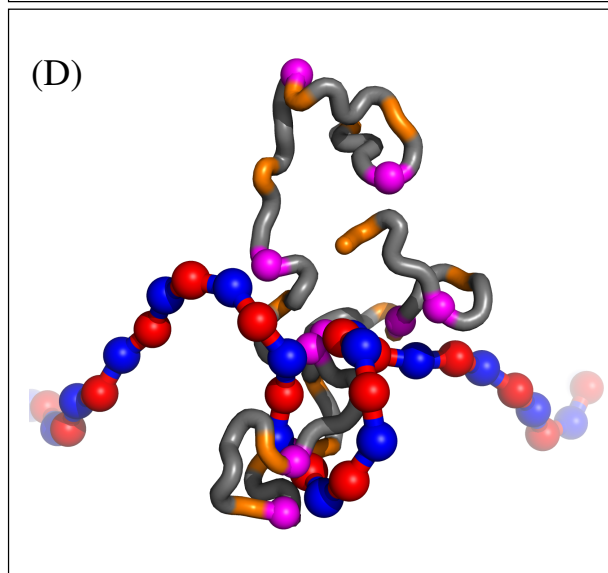

Supplement: Figure S8 — Exploring other EAD-target binding scenarios. The EAD sequences are the same as those in Fig. 1. (A) Simulated EAD binding probability with a hypothetical target in which the surface charges are not evenly distributed but confined to a patch. Two such hypothetical patch partners were considered, both with 12 cations localized on a patch with the same local cation density as the generic target with 32 cations (Fig. S2A) that we have used for most of the simulations. One of the targets (referred to as the positive patch target) contains 12 cations and no anions on the patch whereas the other (referred to as the neutral patch target) contains 12 cations and 12 anions. Plotted here are the simulated binding probabilities for the positive (squares) and neutral (circles) patch targets in either a simulation box of size of (300 Å)3 (black symbols) or (600 Å)3 (blue symbols). (B) A snapshot of an = 10 EAD sequence (tube representation) bound to the neutral patch target. (C) Simulated EAD binding probability with hypothetical disordered (IDP) partners. The EAD sequences and simulation conditions are the same as those in Fig. 1B,C, using a simulation box of size (600 Å)3. During the binding simulations, both the EAD and the hypothetical IDP target were allowed to sample all accessible conformations while the center of mass of the IDP target was kept at a fixed position in the center of the simulation box. We considered a class of such targets, each of which is a chain consisting of 64 alternating cations and anions (32 cations and 32 anions). The adjacent cation and anion are connected by a 5 Å virtual bond with a stiff bond-angle force constant equal to 10.0. Shown here are binding probabilities for four different such IDP targets with equilibrium bond angles that equal, respectively, to 105° (crosses), 120° (diamonds), 135° (squares) and 150° (circles). A general trend of increasing binding with increasing is observed for all four hypothetical IDP targets. Not surprisingly, [file pcbi.1003239.s008.pdf]
