# Supplementary material for: Polycation-π Interactions Are a Driving Force for Molecular Recognition by an Intrinsically Disordered Oncoprotein Family
Source: PLoS Comput Biol. 2013 Sep 26;9(9):e1003239. doi: 10.1371/journal.pcbi.1003239 (PMC3784488; doi:10.1371/journal.pcbi.1003239)
Supplement: Table S1 — Numbers of conformations, or self-avoiding flights, on the simple cubic lattice. Conformational counts as functions of chain length (number of beads) n are obtained by exact enumeration. A chain with n beads has n−1 bonds. Here, is the number of unconstrained conformations; is the number of conformations that have one chain end anchored onto an impenetrable plane (Fig. S5C); and is the number of conformations that have the mid-chain bead [ bead if n is even, bead if n is odd] making a contact with an impenetrable plane (Fig. S5D). (PDF) [file pcbi.1003239.s009.pdf]

n	$\Omega_0(n)$	$\Omega_a^0(n)$	$\Omega_a^m(n)$
4	150	93	85
5	726	409	337
6	3,534	1,853	1,433
7	16,926	8,333	5,937
8	81,390	37,965	25,809
9	387,966	172,265	110,369
10	1,853,886	787,557	486,049
11	8,809,878	3,593,465	2,118,369
12	41,934,150	16,477,845	9,427,777
13	198,842,742	75,481,105	41,662,809
14	943,974,510	346,960,613	186,303,561
15	4,468,911,678	1,593,924,045	828,799,641
16	21,175,146,054	7,341,070,889	3,725,715,541
17	100,121,875,974	33,798,930,541	16,682,103,329
