# Supplementary material for: Polycation-π Interactions Are a Driving Force for Molecular Recognition by an Intrinsically Disordered Oncoprotein Family
Source: PLoS Comput Biol. 2013 Sep 26;9(9):e1003239. doi: 10.1371/journal.pcbi.1003239 (PMC3784488; doi:10.1371/journal.pcbi.1003239)
Supplement: Table S2 — Loop probabilities determined by exact lattice conformational enumeration. Tabulated here are examples (not a complete list) of conformational counts used in Fig. S6. Here one chain end is always in contact with the origin (0,0) of a two-dimensional coordinate system for the impenetrable plane. In this table, the positions on the impenetrable plane where another contact with the chain existed are indicated by the (x,y) coordinates. In the present treatment of our analytical model, values from all combinations of x,y (where x<y) that have nonzero counts for n≤17 were used to estimate the conformational entropic cost of loop formation (Figs. S6 and S7). (PDF) [file pcbi.1003239.s010.pdf]

x,y	R_j	$l = 3$			$l = 4$		$l = 5$
		$n = 4$	$n = 5$	$n = 6$	$n = 5$	$n = 6$	$n = 6$
0,1	1	3	9	38			
0,3	3	1	4	17			
1,2	$\sqrt{5}$	6	24	98			
0,2	2				9	33	
0,4	4				1	4	
1,3	$\sqrt{10}$				8	32	
0,1	1						25
0,3	3						18
0,5	5						1
1,2	$\sqrt{5}$						58
1,4	$\sqrt{17}$						10
2,3	$\sqrt{13}$						20
