# Supplementary material for: Polycation-π Interactions Are a Driving Force for Molecular Recognition by an Intrinsically Disordered Oncoprotein Family
Source: PLoS Comput Biol. 2013 Sep 26;9(9):e1003239. doi: 10.1371/journal.pcbi.1003239 (PMC3784488; doi:10.1371/journal.pcbi.1003239)
Supplement: Text S1 — Experimental and Computational Details and Rationale. (PDF) [file pcbi.1003239.s012.pdf]

Text S1

Supporting Information

for PLoS Comput Biol article

Polycation-π interactions are a driving force for molecular recognition by an intrinsically disordered oncoprotein family

Jianhui Song, [1†] Sheung Chun Ng, [2†] Peter Tompa, [3,4] Kevin A. W. Lee, [2]* and Hue Sun Chan[1]*

[1]Departments of Biochemistry, Molecular Genetics, and Physics, University of Toronto, Toronto, Ontario M5S 1A8 Canada; [2]Division of Life Science, Hong Kong University of Science and Technology, Clear Water Bay, Hong Kong S.A.R., China; [3]VIB Department of Structural Biology, Vrije Universiteit Brussel, Building E, 1050 Brussels, Belgium; and [4]Institute of Enzymology, Hungarian Academy of Sciences, H-1113 Budapest, Hungary.

[*] To whom correspondence may be addressed.
Email: bokaw@ust.hk or chan@arrhenius.med.toronto.edu
[†] These authors contributed equally to this work.

(*Higher-resolution versions of the supporting figures and tables on pages 10-19 of this document are also provided separately as individual files)*

Experimental and Computational Details and Rationale

Experimental details

General aspects of the methodology have been described previously [1,2]. Details that are specific to the present study are as follows.

Plasmids. For the EAD variants that were derived from the mammalian expression vector pSliencer 4.1-CMV neo (Applied Biosystems), pCMVvec contains unique HindIII and BglII sites between the CMV promoter and coding sequence of the ATF1 region present in the EWS/ATF1 oncoprotein except that the ATF1 bZIP domain is replaced with the zta bZIP domain as previously descibed [3]. For construction of EAD mutants, HindIII/BglII ended synthetic DNA fragments were obtained by commercial gene synthesis (TOP Gene Technologies, Montreal, QC, Canada) and directly inserted into pCMVvec digested with HindIII/BglII. All proteins also contained the KT3 monoclonal epitope PPPEPET [4] at the C-terminus adjacent to the zta bZIP domain.

Proteins. The complete amino acid sequences of all EAD mutants are shown in Fig. S1. 10Yn protein contains an EAD peptide identical to EAD N-terminal residues 1-66 except that the position of four of the ten Ys present is exchanged with nearby residues to give approximately even spacing of Y residues (Fig. 1 in the main text and Fig. S1A). For 9Yn-4Yn the corresponding number of Y residues in 10Y are substituted with prevalent residues in EAD (namely Ala, Gly, Thr, Ser or Gln) to maintain the overall composition and have regularly spaced Ys located in the middle of the peptide at the same density as 10Yn. 5Fn and 5Wn proteins correspond to 5Yn with all Ys replaced by F and W respectively. 5Y protein corresponds to native EAD1-66 with alternate Y residues replaced with prevalent residues in EAD. 7Yn/2 and 7Yn/4 are related to 7Yn and contain seven evenly spaced Ys with linear Y density (given by the distance between the first and last Ys) of approximately half (7Yn/2) and one quarter (7Yn/4) of that in 7Yn. Extra sequences present in 7Yn/2 and 7Yn/4 were derived from 7Yn (minus Ys) to maintain overall composition. Other EAD mu-

tants shown in Fig. S1 are 10Y3D, 5Y5D, 10Y3R, 10Y5R, 8YΔD, 8Y2RΔD and 6YΔD.

Transactivation assays and Western blotting. Activity values were corrected for background activity determined by including the EAD-negative protein ZΔE in transfections. In cases where EAD mutations resulted in significant changes in protein levels, protein expression in vivo was normalized by using different amounts of plasmid for transfection. Luciferase assays were performed at 40 h post-transfection. Western blotting using primary antibody KT3 [4] and alkaline phosphatase conjugated anti-mouse secondary antibody (DAKO) were as previously described [5].

Rationale and computational details of the chain simulation model

Intra-EAD and EAD-target interaction potentials. As outlined in Method of the main text, our chain simulation model describes binding of various EAD sequences (Fig. S1) with a generic target (binding partner) which is a sphere of radius R_p = 16.0Å. The total potential energy of the model system $E_\mathrm{T} = E_\mathrm{intrachain} + E_\mathrm{chain\text{-}target}$ is the sum of the intramolecular energy $E_\mathrm{intrachain}$ and the intermolecular chain-target energy $E_\mathrm{chain\text{-}target}$, where

$$\begin{aligned} E_\mathrm{intrachain} = & \sum_{i=1}^{n-2} \varepsilon_\theta (\theta_i - \theta_0)^2 \\ & + \sum_{i=1}^{n}\sum_{j=1}^{n} \Bigg\{ \varepsilon_\mathrm{ex} \left(\frac{r_{\mathrm{rep},ij}}{r_{ij}}\right)^{12} \\ & + \varepsilon_{\mathrm{h}\varphi} \kappa_i \kappa_j \exp\left[-\left(\frac{r_{ij}}{2r_0}\right)^2\right] \\ & + \varepsilon_{\mathrm{c}\pi}^{ij} \left[\left(\frac{\sigma_{\mathrm{c}\pi}}{r_{ij}}\right)^{12} - \left(\frac{\sigma_{\mathrm{c}\pi}}{r_{ij}}\right)^{6}\right] \\ & + \frac{1}{4\pi\epsilon_0\epsilon_\mathrm{d}} \frac{q_i q_j}{r_{ij}} \exp\left(-\frac{r_{ij}}{\lambda_\mathrm{D}}\right) \Bigg\}. \end{aligned} \quad (\mathbf{S1})$$

Here n is the number of residues in the EAD sequence; i,j label residue positions; θ_i is the ith virtual bond angle (in radian), $\theta_0 = \pi/2$ is the reference virtual bond angle, r_{ij} is the spatial distance between the ith and jth residues. ε_θ, ε_ex, and $\varepsilon_{\mathrm{h}\varphi}$ are the interaction strengths for bond angle, excluded volume and hydrophobic interactions, and are set to 1.0, 1.0, and –3.0 $k_\mathrm{B}T$ respectively (where k_B is Boltzman constant and T is absolute temperature). The range of excluded volume repulsion $r_{\mathrm{rep},ij}$ = 5.0 Å if both i and j are charged residues; otherwise $r_{\mathrm{rep},ij}$ = 4.0 Å for all other residue pairs as in our previous protein chain models [6,7]. κ_i = 1 for hydrophobic residues, κ_i = 0 otherwise; and $2r_0 = b$ = 3.8Å sets the range of the hydrophobic interactions. $\varepsilon_{\mathrm{c}\pi}^{ij}$ is the cation-π interaction strength that depends on the aromatic residue and is nonzero only when (i,j) is a (cation, π) or (π, cation) pair; and $\sigma_{\mathrm{c}\pi}$ is set to 4.0 Å. In the electrostatic (last) term, q_i (= 0 or ±1) is the charge of residue i; ϵ_0 is vacuum permittivity, ϵ_d = 40 is the dielectric constant; and $\lambda_\mathrm{D}$is the screening length, which we set to 10.0Å to mimic physiological conditions. The EAD-target interaction is given by

$$\begin{aligned} E_\mathrm{chain-target} = & \sum_{i=1}^{n} \varepsilon_\mathrm{ex} \left(\frac{r_0 + R_\mathrm{p}}{r_{\mathrm{c}i}}\right)^{12} \\ & + \sum_{i=1}^{n}\sum_{k=1}^{N_\mathrm{c}} \Bigg\{ \varepsilon_{\mathrm{c}\pi}^{iv} \Bigg[\left(\frac{\sigma_{\mathrm{c}\pi}}{r_{iv}}\right)^{12} \\ & - \left(\frac{\sigma_{\mathrm{c}\pi}}{r_{iv}}\right)^{6}\Bigg] \\ & + \varepsilon_\mathrm{ex} |q_i q_v| \left(\frac{r_\mathrm{rep}}{r_{iv}}\right)^{12} \\ & + \frac{1}{4\pi\epsilon_0\epsilon_\mathrm{d}} \frac{q_i q_v}{r_{iv}} \exp\left(-\frac{r_{iv}}{\lambda_\mathrm{D}}\right) \Bigg\}, \end{aligned} \quad (\mathbf{S2})$$

which is a sum of energy terms for EAD-partner excluded volume, cation-π, excluded volume between charged residues (r_rep = 5.0 Å), and electrostatic interactions (in the order given in the above equation). The index v labels the charges (q_vs) on the binding partner, r_{iv} is the distance between residue i in the EAD and charge v on the partner, and $r_{\mathrm{c}i}$ is the distance between the center of the partner and EAD residue i.

The model globular target. As outlined in the main text, the generic target is modeled by a sphere of radius R_p with positive and negative charges embedded on its surface (Fig. S2A). The EAD sequences are modeled as C_α chains (Fig. S2B). Taking a simple, minimalist approach, we

assume that the generic target is electrically neutral with equal numbers of positive and negative charges, and that the charges are evenly distributed on the surface of the generic target. We employed the "Golden Section Spiral Algorithm" [8,9] implemented in MATLAB (MathWorks, Natick, Massachusetts) to construct essentially even distributions for the charges. Most of the EAD binding simulations reported in this work – unless noted specifically otherwise – are for a generic target with 32 positive charges and 32 negative charges, wherein the distances between two nearest neighboring charges of the same sign and of opposite signs are, respectively, 9.4 Å and 5.1 Å. These distances were designed to mimic the charge distribution of RNA polymerase II subunits Rpb4/Rpb7 (PDB ID: 2C35) [10], for which the shortest distances between positive-positive, negative-negative, and positive-negative pairs are 9.4 Å (Fig. S2C), 7.7 Å, and 5.7 Å, respectively.

Model EAD chains. The C_α chain model for an EAD may be envisioned as a string of beads wherein the distance between the centers of two adjacent beads is equal to the C_α–C_α virtual bond length b = 3.8Å (Fig. S2B). Similar coarse-grained C_α models have provided much biophysical insight into protein folding and dynamics (see, e.g., [7] for a recent review). In the present EAD chain model, we set the reference virtual bond angle θ_0 in Eq. **(S1)** to π/2 radian (90°), which coincides approximately with the peak value of the distribution of virtual bond angles in the Protein Data Bank (PDB) [11] but is somewhat smaller than the 106.3 ° [12] or 105 ° [13] used in other C_α chain models for proteins. To allow for more chain flexibility and efficient sampling of a large number of possible EAD conformations, we adopted a weak interaction strength ε_θ = 1.0$k_{\rm B}T$ for the virtual bond angle term in Eq. **(S1)**. Consequently, every virtual bond angle θ_i can sample a range from π/4 (45º) to 3π/4 (135º) quite freely because it entails an energetic cost of at most ~0.6$k_{\rm B}T$.

Types of interaction in the model. Both the intra-EAD and EAD-partner interactions [Eqs. **(S1)** and **(S2)** respectively] are dependent upon the EAD sequence. Pairwise interactions between amino acid residues (represented by their C_α positions) depend on whether they are aromatic (Y, F, W), hydrophobic (A, V, L, I, M, W, F, Y, P) — which include the aromatics [14], charged (D, E, R, K), or polar (N, C, Q, G, H, S, T). An EAD chain is considered to be bound to the generic target if at least one aromatic residue along the EAD sequence is spatially within a capture radius $R_{\rm c}$ = 6.0Å from a target cation (Fig. S2D). In the present model, the N- and C-termini of the EAD chain carry a positive and a negative charge respectively. These charges participate in intra-EAD and EAD-target electrostatic interactions, but the N-terminal positive charge does not engage in cation-π interactions.

Strengths of cation-π contacts in the model. In the present modeling setup, the total interaction between an aromatic residue and a cation is the sum of one of the 12-6 Lennard-Jones potentials in Fig. S2E and a general excluded volume term, which is equal to $\varepsilon_{\rm ex}(r_{{\rm rep},ij}/r_{ij})^{12}$ for an intra-EAD cation-π interaction [Eq. **(S1)**] and $\varepsilon_{\rm ex}[(r_0+R_{\rm p})/r_{{\rm c}i}]^{12}$ for an EAD-target cation-π interactions [Eq. **(S2)**]. The effects of these two general excluded volume terms are similar and are relatively small, each amounting to a decrease of well depth by ≈ 0.16 kcal/mol relative to the potentials in Fig. S2E. The total intra-EAD cation-π potentials in Fig. 1B are practically identical to the corresponding total EAD-target cation-π potentials along the radial direction of the target. As discussed in the main text, the well depths of our model cation-π interactions (Fig. 1B) are in line with published estimates of cation-π potentials of mean force in aqueous environments with well depths ≈ –3.0 to –5.5 kcal/mol [15–17]. The differences in well depth among our model cation-Y, cation-F, and cation-W interactions were designed in accordance with PDB statistics. We relied largely on the PDB cation-π contact frequencies compiled by Gallivan and Dougherty [15] in this regard. Because the PDB structures were determined in aqueous environments, PDB statistics are more directly relevant to the aqueous cation-π potentials of mean force we aim to model than experimental and theoretical data on cation-π interactions in the absence of solvation effects [18]. In the dataset considered by Gallivan and Dougherty [15], the frequencies of R, K, Y, F, and W are, respectively, p(R) = 10,919, p(K) = 13,446, p(Y) = 8,309, p(F) = 9,162, and p(W) = 3,412; and the frequencies of R-Y, R-F, R-W, K-Y, K-F, and K-W contacts are, respectively, p(R-Y) = 749, p(R-F) = 630, p(R-W) = 609, p(K-Y) = 438, p(K-F) = 285, and p(K-W) = 283 (Table 1 of [15]). Using a simple formulation for statistical potential [19] and $k_{\rm B}T$ ≈ 0.6 kcal/mol for T ≈ 300K, the difference in cation-Y and cation-F contact energy (former minus latter) may be es-

timated as $-k_BT\ln\{[p(\text{R-Y})/p(\text{R-F})][p(\text{F})/p(\text{Y})]\}$ = –0.16 kcal/mol for an R cation and as $-k_BT \times \ln\{[p(\text{K-Y})/p(\text{K-F})][p(\text{F})/p(\text{Y})]\}$ = –0.32 kcal/mol for a K cation. These energy differences are consistent with the –0.07 to –0.37 kcal/mol range of well depth differences between cation-Y and cation-F interactions we adopted in Fig. 1B. Similarly, the difference in cation-Y and cation-W contact energy (former minus latter) may be estimated as $-k_BT\ln\{[p(\text{R-Y})/p(\text{R-W})]\times[p(\text{W})/p(\text{Y})]\}$ = +0.41 kcal/mol for an R cation and $-k_BT\ln\{[p(\text{K-Y})/p(\text{K-W})][p(\text{W})/p(\text{Y})]\}$ = +0.27 kcal/mol for a K cation. These energy differences are quite similar to the +0.42 kcal/mol well depth difference between cation-Y and cation-W in Fig. 1B. In the PDB analysis of Crowley and Golovin [17], W is also seen to interact significantly stronger with R than Y or F for protein complexes and homodimers, but the trend is less clear for the interaction of W, Y, or F with K (Table III in [17]).

Hydrophobic and electrostatic interactions. The functional forms for the hydrophobic and electrostatic potentials in the present model (Fig. S2F) are similar to those we used in previous coarse-grained modeling studies [20,21]. For most of the present simulations, we used a hydrophobic interaction strength $\varepsilon_{h\varphi} = -3.0\ k_BT$ and a dielectric constant ϵ_d = 40. We chose an ϵ_d value intermediate between the dielectric constant ≈78.5 for bulk water and ~ 2 – 4 for the interior of a folded protein [22] because physically both the intra-EAD and EAD-target electrostatic interactions take place in an aqueous environment (not the interior of a folded protein) but with significant effective local protein (IDP) concentration.

Varying the hydrophobic interaction strength. To assess the robustness of our model predictions and to better delineate the conditions for the validity of the predictions, we have conducted control simulations using alternative values of $\varepsilon_{h\varphi}$ and ϵ_d. We found that strengthening the hydrophobic interaction strength from $\varepsilon_{h\varphi} = -3.0\ k_BT$ (well depth ≈0.25 kcal/mol) to $\varepsilon_{h\varphi} = -7.0\ k_BT$ (well depth ≈ 0.9 kcal/mol) while keeping other modeling parameters unchanged did not have much effect on the binding of the 4Yn – 10Yn sequences in Fig. 1B. However, binding became very weak when hydrophobic interaction was strengthened to $\varepsilon_{h\varphi} = -13.0\ k_BT$ (well depth ≈2.0 kcal/mol) because in that case the aromatics would interact strongly with other aromatics and/or other hydrophobic residues and are sequestered in the interior of compact conformations instead of undergoing cation-π interactions with the target.

Varying the electrostatic interaction strength. We have also considered two alternative ϵ_d values while keeping other modeling parameters unchanged. First, application of the distance-dependent dielectric constant of Jha and Freed, viz., $\epsilon_d(r) = (\epsilon_d^b - \epsilon_d^0)[(sr)^2 + 2sr + 2]\exp(-sr/2)$, where ϵ_d^b = 78.5 is the dielectric constant of bulk water, ϵ_d^0 = 1.77 and s = 0.274 [23] led to an even shallower attractive well for the electrostatic interactions than ϵ_d = 40. Using this $\epsilon_d(r)$ led only to small changes to the simulated binding probabilities for the 4Yn –10Yn sequences in Fig. 1B. Second, we tested ϵ_d = 20. This ϵ_d value led to a deeper attractive well of ≈ 1.5 kcal/mol for the electrostatic interactions (blue dashed curve in Fig. S2F), which is ≈1.5/3.6 ~ 40% of the cation-π well depth in our model. This ratio of ~40% is relevant because a quantum mechanical calculation of the methylammonium-acetate and the methyl- ammonium-benzene potentials of mean force in water by Gallivan and Dougherty suggests the same ratio (−2.2 kcal/mol / −5.5 kcal/mol = 40%) of well depths between typical salt-bridge and cation-π interactions [16]. For a test set of EAD sequences that include the highly charged 10Y5R, 10Y3R, and 10Y3D in Fig. 3 and Fig. S1, the simulated binding probabilities using ϵ_d = 20 are quite similar to the corresponding probabilities simulated using ϵ_d = 40. Specifically, the simulated P_b values of 4Yn, 5Yn, 6Yn, 7Yn, 8Yn, 9Yn, and 10Yn are, respectively, 0.011, 0.025, 0.057, 0.109, 0.196, 0.297, and 0.427 for ϵ_d = 40 (data plotted in Fig.1B) and are 0.008, 0.029, 0.059, 0.098, 0.165, 0.312, and 0.471 for ϵ_d = 20. For the charged sequences 10Y3D, 5Y5D, 10Y3R, 10Y5R, 8YΔD, 6YΔD, and 8Y2RΔD, the simulated P_b values are, respectively, 0.370, 0.003, 0.070, 0.005, 0.057, 0.017, and 0.019 for ϵ_d = 40 and 0.386, 0.003, 0.084, 0.006, 0.049, 0.021, and 0.025 for ϵ_d = 20. Taken together, results from our control simulations suggest that the trends predicted by our model should be robust inasmuch as the EAD does not undergo a hydrophobic collapse and that cation-π interactions are significantly stronger than salt-bridge interactions as stipulated by theoretical considerations [16].

Conformational sampling. Monte Carlo sampling of EAD conformations was conducted at T = 300K in a 600Å×600Å×600Å simulation box with periodic boundary conditions and the target fixed at its center. The EAD is defined to be bound if at least one aromatic on it is within a capture radius R_c = 6.0Å from a target cation (Fig. S2D). Binding probabilities were computed accordingly. Four types of chain moves were used for conformational sampling with equal attempt probabilities: rigid rotations, pivot moves, kink-jumps [24,25] and translation moves. In a translation move, a random direction and a random distance ≤ 1Å is selected. This random vector of displacement is then applied to every bead of the chain. All four types of attempted chain moves were accepted or rejected by applying the standard Metropolis criterion [26] to the total energy $E_T = E_{\text{intrachain}} + E_{\text{chain-target}}$ in Eqs. **(S1)** and **(S2)**. The acceptance rate is ~50%. In a typical binding simulation with the generic target, ~20 million attempted chain moves were used for initial equilibration and data collection was conducted during the subsequent ~80 million attempted chain moves. For each of the binding simulation with an IDP target (see below), ~40 million attempted chain moves were used for initial equilibration and ~40 million subsequent attempted chain moves were used for data collection. Because the excluded volume and hydrophobic terms are of short spatial range, a 10.0Å cutoff was applied to the ε_{ex} and $\varepsilon_{\text{h}\varphi}$ terms in Eq. **(S1)** and a $2r_0 + R_p$ = 19.8Å cutoff was applied to the ε_{ex} term in Eq. **(S2)** for computational efficiency.

Radii of gyration of the model EAD chains. For the 4Yn–10Yn EAD sequences in Fig. 1, the simulated mean radii of gyration (using $\varepsilon_{\text{h}\varphi}$ = –3.0 k_BT and ϵ_d = 40) for unbound EADs are ~21Å, whereas the simulated mean radii of gyration for bound EADs vary slightly from ≈22.5Å for 4Yn to ≈20.3Å for 10Yn. For the EAD sequences with positively charged Rs in Fig. 3 of main text, intra-EAD cation-π contacts lead to more compact unbound conformations. For 8Y2RΔD (with two Rs), 10Y3R (with three Rs), and 10Y5R (with five Rs), the simulated mean radii of gyrations are, respectively, 17.6, 15.6, and 12.1Å. In contrast, the simulated mean radii of gyration for 10Y3D and 8YΔD (with no R) are 21.7 and 21.1Å, respectively, which are practically identical to the ~21Å mentioned above for the 4Yn–10Yn sequences in Fig. 1.

Matching chain simulation results with experimental data. The chain simulation model described above was applied to analyze the experimental activity measurements reported in the main text. In addition, the model was also used to re-analyze earlier experimental activity data [1] (Fig. S3), to address the similarities and differences in the binding properties of monomer and dimer EADs (Fig. S4), to motivate our analytical model (discussion below and Figs. S5–S7), and to study possible association of EAD with IDP targets (Fig. S8) including the RGG3 sequence in the Ewing's sarcoma RNA-binding domain [27,28]. Although how precisely real EAD binding triggers specific functional events is not known, we recognize that the energetics of oncogenic EAD processes is unlikely to comprise solely of the interactions included in our model. For instance, a certain energetic contribution can be associated with a specific functional event, similar to the contact energy E_b in the polyelectrostatics model [29]. In the approach adopted here, we assume as a first approximation that this unknown energy (let us denoted it as $E_{\text{b,EAD}}$ for the discussion at hand) is a constant irrespective of the bound EAD conformation. Because we did not include such an energy in our model, the binding free energy $\Delta G_b = -k_BT\ln[P_b/(1 - P_b)]$ in our model (Fig. 2D and Fig. S4) was computed without regard to $E_{\text{b,EAD}}$. In view of this limitation, the ΔG_b values in the present work were used to address only relative, but not absolute, activities of real EAD, because the true binding free energy could have been $\Delta G_b + E_{\text{b,EAD}}$.

Rationale and constructional details of the analytical model

Overall goal, strengths and limitations. The analytical model in this work [Eq. **(1)** in the main text] was developed as a complement to the chain simulation. Our goal in developing the analytical model is to provide further insights into the trends observed in simulations and experiments. The analytical model addresses multisite EAD binding by considering the balance among the energetic contributions from cation-π contacts, translational and conformational entropies of the EAD as well as intra-EAD and EAD-target excluded-volume effects. For simplicity, hydrophobic and electrostatic interactions are not incorporated in the present analytical model. As a model for chain behavior, the analytical model

lacks an explicit representation of the polymer chain and thus is less accurate than the chain simulation model. Despite its reliance on approximations, the analytical model is valuable because it offers conceptual clarity and computational efficiency. Its tractability allows for efficient exploration of model parameters and, therefore, a more comprehensive assessment of the robustness of the model's predictions.

Conformational entropic effects of EAD binding. Multisite binding of EAD entails large changes in its conformational ensemble upon binding. Therefore, unlike the mean-field polyelectrostatic model for the Sic1-Cdc4 system [29] that assumes no significant changes in the conformational shape of the IDP ligand upon binding, we now need to estimate the change in conformational entropy upon binding of an EAD to its target. As outlined in the main text, in order to afford a rudimentary account of excluded volume effects on conformational freedom, we adopted exact lattice enumeration to assist in the necessary entropy estimations. Exact enumeration of lattice conformations is a powerful and versatile technique that has contributed to fundamental advances in polymer physics and in the study of protein structure and stability [30–32].

A simple model of the EAD sequence. All ingredients of the analytical model have been introduced in the main text. Again, for simplicity, the present formulation of the analytical model considers only EAD sequences with N_π equally spaced aromatics separated by the same number of amino acid residues (k bonds between two successive aromatics), as illustrated by Fig. S5A. If necessary, this restriction can be relaxed as the model can readily be generalized to tackle EAD sequences with any set of aromatic positions. In our analytical model, the aromatic positions along the EAD define a set of possible loops of EAD chain segments by having two or more EAD aromatics contacting the cations on the target. The conformational entropy of such a loop is determined by the length l of the loop and the distance R_j between the two cation-aromatic contacts. We used the geometry of the generic target in the chain simulation model to determine the distribution $n_{\rm c}(R_j)$ for R_j (Fig. S5B).

Lattice estimation of conformational entropy. Exact enumeration of conformations in the presence of an impenetrable infinite planar surface [33] was utilized to provide a general approximation of the loop conformational entropy that we can readily apply to targets with different geometries. We first obtained the numbers of conformations (self-avoiding flights) configured on a simple cubic lattice that are (i) subject to no constraint, i.e., it is free to configure on an infinite lattice subject only to the condition that it cannot intersect itself, (ii) constrained to have one end of the chain contacting the impenetrable plane, as illustrated in Fig. S5C, and (iii) constrained to have the middle of the chain contacting the impenetrable plane, as illustrated in Fig. S5D. We denote the number of such conformations, as a function of chain length n, by $\Omega_0(n)$, $\Omega_{\rm a}^0(n)$, and $\Omega_{\rm a}^{\rm m}(n)$, respectively. These conformational counts are given in Table S1 for n = 4 to n = 17. The quantity $\Omega_0(n)$ has been studied extensively before. The $\Omega_0(n)$ counts in Table S1 are consistent with an early $n \le 16$ enumeration by Sykes [34] and a more recent enumeration by Clisby et al [35]. More specifically, our $\Omega_0(n)$ is equivalent to the coefficient for x^{n-1} in Eq. 2.1 of [34]. $\Omega_0(17)$ was also provided in this reference but the coefficient 100,117,875,366 for x^{16} was incorrect. Our $\Omega_0(n)$ corresponds to the variable c_{n-1} in Table A5 of [35], which provides $\Omega_0(n)$ for $n \le 31$. Note, however, that the variable n in Clisby et al [35] is the number of bonds and thus is equivalent to our n – 1.

The quantity $\ln[\Omega_{\rm a}^0(n)/\Omega_0(n)]$ represents the change in conformational entropy, in units of $k_{\rm B}$, upon bringing a free, unconstrained chain to the vicinity of the impenetrable surface and making a first contact with the surface at a chain end. Likewise, $\ln[\Omega_{\rm a}^{\rm m}(n)/\Omega_0(n)]$ represents a similar entropy change but the first contact with the plane is made at mid-chain. For random flights, the corresponding conformational entropy change scales as – (ln n)/2 irrespective of which point along the chain makes the contact with the plane [36]. However, for self-avoiding flights, the difference between $\ln[\Omega_{\rm a}^{\rm m}(n)/\Omega_0(n)]$ and $\ln[\Omega_{\rm a}^0(n)/\Omega_0(n)]$ is significant and increases with n (Fig. S5E). In the present formulation of the analytical model, we used $\ln[\Omega_{\rm a}^{\rm m}(n)/\Omega_0(n)]$ to provide a general approximation for the conformational entropy change upon the formation of the first chain-plane contact [see Eq. **(1)** of main text]. The reason for this choice is that for the chain lengths we studied, a chain-plane contact is more likely to be sufficiently far away from the chain ends to be better represented by a mid-chain contact rather than a chain-end contact. Accordingly, in binding free energy calculations using Eq. **(1)** of main text, $\ln[\Omega_{\rm a}^{\rm m}(n)/\Omega_0(n)]$ was determined using the data in Table

S1 for $n \leq 17$ and estimated for $n > 17$ by extrapolating the fitting equation for $\ln[\Omega_{\rm a}^{\rm m}(n)/\Omega_0(n)]$ provided in the caption for Fig. S5.

After the first EAD-target contact has been made, EAD loops can form on the target surface, leading to further reduction in conformational entropy. We estimated such entropy reduction by enumerating $\Omega(l, R_j|n)$, which is the number of conformations with one chain end anchored to the impenetrable surface while a loop of length l is formed by a second contact at a distance R_j from the anchored chain end (Fig. S6, top left drawing). Examples of such conformational counts are provided in Table S2; and a complete listing of $\Omega(l = n-1, R_j|n)$ counts for $n = 17$ is included in Table S3 to illustrate our method. Values for $\ln[\Omega(l, R_j|n)/\Omega_{\rm a}^{\rm m}(n)]$ from $n = 4$ through $n = 17$ were then grouped by loop length l and plotted in Fig. S6. Recognizing that $\ln[\Omega(l, R_j|n)/\Omega_{\rm a}^{\rm m}(n)]$ for a given l is not very sensitive to chain length n, we obtained quadratic fits for $\ln[\Omega(l, R_j)/\Omega_{\rm a}^{\rm m}]$ in the form of $-a(l)[R_j - b(l)]^2 + c(l)$ for $l \leq 16$ (caption of Fig. S6). We then extrapolated the fitting parameters $a(l)$, $b(l)$, and $c(l)$ for $l > 16$ (Fig. S7A,B,C) to obtain the necessary $\Omega(kl_i, R_j|n)/\Omega_{\rm a}^{\rm m}(n)$ values (now approximated as independent of n) that enter Eq. **(1)**. It should be noted here that R_j was measured in units of lattice bond length in the enumeration data, and that the lattice bond length is taken to be equivalent to the C_α–C_α virtual bond length b = 3.8Å in our analysis. It follows that the R_j values in the $n_{\rm c}(R_j)$ distributions in Fig. S5B have to be converted to lattice units ($R_j \rightarrow R_j/b = R_j/3.8$Å) before they enter Eq. **(1)**.

Robustness of the predicted binding free energies. For the EAD sequences studied in this work, we found that the binding free energy is not very sensitive to the functional form of $\ln[\Omega(l, R_j)/\Omega_{\rm a}^{\rm m}]$ for large l. To evaluate this sensitivity, we have compared binding free energies calculated using the above procedure and one that used exact enumeration $\ln[\Omega(l, R_j)/\Omega_{\rm a}^{\rm m}]$ for $l \leq 16$ but substituted $\ln[\Omega(l, R_j)/\Omega_{\rm a}^{\rm m}]$ with the random-flight expression $\ln[\Omega(l, R_j|n)/\Omega_0(n)] = (3/2)\ [\ln(3/2\pi) - \ln(l) - R_j^2/l]$ for $l > 16$. [The latter expression follows from the random-flight probability $(3/2\pi)^{3/2}\exp(-3R_j^2/2l)$ for a chain of length l starting from the origin and ending at a position that is at a distance R_j from the origin.] Despite the appreciable difference between the two entropy expressions for large l (Fig. S7D), the calculated binding free energies using the two different schemes are nearly identical for the set of sequences tested in Fig. S7E.

Energetic and entropic components of the binding free energy. $\Delta G_{\rm b}$ can readily be expressed as a sum of an energy and an entropy, with the binding energy (enthalpy) given by

$$\Delta E_{\rm b} = \frac{E_{{\rm c}\pi}}{Q_{\rm b}'}\left\{N_\pi + \sum_{\{l_i\}}(n_{\rm loop}+1) \times \prod_i e^{-E_{{\rm c}\pi}/k_{\rm B}T} \sum_j n_{\rm c}(R_j) \times \left[\frac{\Omega(kl_i, R_j|n)}{\Omega_{\rm a}^{\rm m}(n)}\right]\right\} \qquad \textbf{(S3)}$$

where $n_{\rm loop} = \sum_i 1$ is the number of loops and $n_{\rm loop} + 1$ is the number of cation-π contacts, and

$$Q_{\rm b}' = N_\pi + \sum_{\{l_i\}}\prod_i e^{-E_{{\rm c}\pi}/k_{\rm B}T} \times \sum_j n_{\rm c}(R_j)\left[\frac{\Omega(kl_i, R_j|n)}{\Omega_{\rm a}^{\rm m}(n)}\right]. \qquad \textbf{(S4)}$$

After $\Delta E_{\rm b}$ has been determined, the binding entropy $\Delta S_{\rm b}$ can be calculated using the standard relation

$$T\Delta S_{\rm b} = \Delta E_{\rm b} - \Delta G_{\rm b}\,, \qquad \textbf{(S5)}$$

where $\Delta G_{\rm b}$is given by Eq. **(1)** in the main text. These expressions were used to compute the $\Delta E_{\rm b}$ and $T\Delta S_{\rm b}$ values in the inset of Fig. 2A.

Possible interference among multiple Ys interacting with same cation

Our present simulation model is seen to overestimate the affinity of 5YP in Fig. 4 of the main

text. The experimental activity of 5YP is approximately the same as that of 10Yn, but the simulated P_b for 5YP is more than double that of 10Yn. To address this mismatch, we note that in the present formulation of our model, two sequentially adjacent Ys are assumed to be able to interact strongly and simultaneously with the same cation as if the two Ys were far apart along the sequence and interacting with different cations. But in reality, the two adjacent Ys would most likely interfere with each other, resulting in weakened individual interactions with the same cation, as cation-π interactions are strongly orientation dependent [37]. This issue did not arise for the other EAD sequences we have simulated in this work because the individual Ys are well separated in those sequences. As a first attempt to explore this issue, we have performed additional simulations using a modified model in which the well depth for an individual cation-Y interaction is reduced from the full strength of 3.58 kcal/mol (Fig. 1B) when the given cation is interacting with two or more Ys. By considering a class of such models we found that if the reduced individual well depth is 2.86 kcal/mol for multiple Y contacting the same cation (representing a ≈20% reduction), the simulated binding probability for 5YP (P_b = 0.46) would be similar to that for 10Yn (P_b = 0.43). To account for the behavior of the 5YP sequence in the analytical model, we considered a model with N_π = 5 and k = 12, but with $E_{c\pi}$ replaced by an energy $E_{c\pi}^{(2)}$ for the combined cation-π interaction energy when a pair of Y's contact a cation simultaneously. If we take $E_{c\pi}^{(2)} = 2E_{c\pi} = 2(-3.5k_BT) = -7.0\,k_BT$, 5YP is predicted by our analytical model to bind much more tightly ($\Delta G_b = -11.7k_BT$) than 10Yn ($\Delta G_b = -3.2k_BT$). However, if $E_{c\pi}^{(2)} = -5.2\,k_BT$ (≈74% of $2E_{c\pi}$), the corresponding binding free energy $\Delta G_b \approx -3.1k_BT$ for 5YP is similar to that for 10Yn as observed in the activity experiments. $E_{c\pi}^{(2)}$ is slightly weaker for the open circle data point that was included in Fig. 4 of the main text as an example ($E_{c\pi}^{(2)} = -5.1\,k_BT$, ≈73% of $2E_{c\pi}$), resulting in slightly weaker binding with $\Delta G_b \approx -2.6k_BT$. These considerations indicate that an interference effect between two aromatics contacting the same cation that amounts to a ~20–30% moderate reduction in the individual cation-π interaction strengths would be sufficient to provide a quantitative account for the experimental activity of 5YP in the context of a polycation-π model.

Supporting References

1. Feng L, Lee KAW (2001) A repetitive element containing a critical tyrosine residue is required for transcriptional activation by the EWS/ATF1 oncogene. *Oncogene* 20:4161-4168.
2. Ng KP, Cheung F, Lee KAW (2010) A transcription assay for EWS oncoproteins in Xenopus oocytes. *Protein Cell* 1: 927–934.
3. Ribeiro A, Brown A, Lee KAW (1994) An in vivo assay for members of the CREB family of transcription factors. *J Biol Chem* 49:31124-31128.
4. MacArthur H, Walter G (1984). Monoclonal antibodies specific for the carboxy terminus of simian virus 40 large T antigen. *J Virol* 52:483–491.
5. Ng KP, Potikyan G, Savene ROV, Denny CT, Uversky VN, Lee KAW (2007) Multiple aromatic side chains within a disordered structure are critical for transcription and transforming activity of EWS family oncoproteins. *Proc Natl Acad Sci USA* 104:479-484.
6. Kaya H, Chan HS (2003) Solvation effects and driving forces for protein thermodynamic and kinetic cooperativity: How adequate is native-centric topological modeling? *J Mol Biol* 326:911-931.
7. Chan HS, Zhang Z, Wallin S, Liu Z (2011) Cooperativity, local-nonlocal coupling, and nonnative interactions: Principles of protein folding from coarse-grained models. *Annu Rev Phys Chem* 62:301-326.
8. Rakhmanov EA, Saff EB, Zhou YM (1994) Minimal discrete energy on the sphere. *Math Res Lett* 1:647-662.
9. Saff EB, Kuijlaars, ABJ (1997) Distributing many points on a sphere. *Mathematical Intelligencer* 19: 5-11.
10. Meka H, Werner F, Cordell SC, Onesti S, Brick P (2005) Crystal structure and RNA binding of the Rpb4/Rpb7 subunits of human RNA polymerase II. *Nucl Acids Res* 33:6435-6444.

11. Gregoret LM, Cohen FE (1991) Protein folding. Effect of packing density on chain conformation. *J Mol Biol* 219:109-122.
12. Levitt M (1976) Simplified representation of protein conformations for rapid simulation of protein folding. *J Mol Biol* 104:59-107.
13. Brown S, Fawzi NJ, Head-Gordon T (2003) Coarse-grained sequences for protein folding and design. *Proc Natl Acad Sci USA* 100:10712-10717.
14. Zarrine-Afsar, A, Wallin S, Neculai AM, Neudecker P, Howell PL, Davidson AR, Chan HS (2008) Theoretical and experimental demonstration of the importance of specific nonnative interactions in protein folding. *Proc Natl Acad Sci USA* 105:9999-10004.
15. Gallivan JP, Dougherty DA (1999) Cation-π interactions in structural biology. *Proc Natl Acad Sci USA* 96:9459-9464.
16. Gallivan JP, Dougherty DA (2000) A computational study of cation-π interaction vs salt bridges in aqueous media: implications for protein engineering. *J Am Chem Soc* 122:870-874.
17. Crowley PB, Golovin A (2005) Cation-π interactions in protein–protein interfaces. *Proteins* 59:231-239.
18. Wu R, McMahon TB (2008) Investigation of cation-π interactions in biological systems. *J Am Chem Soc* 130:12554-12555.
19. Miyazawa S, Jernigan RL (1985) Estimation of effective interresidue contact energies from protein crystal structures: Quasi-chemical approximation. *Macromolecules* 18: 534-552.
20. Zhang Z, Chan HS (2010) Competition between native topology and nonnative interactions in simple and complex folding kinetics of natural and designed proteins. *Proc Natl Acad Sci USA* 107:2920-2925.
21. Zarrine-Afsar A, Zhang Z, Schweiker KL, Makhatadze GI, Davidson AR, Chan HS (2012) Kinetic consequences of native state optimization of surface-exposed electrostatic interactions in the Fyn SH3 domain. *Proteins* 80:858-870.
22. Leach AR (1996) *Molecular Modeling: Principles and Applications*. (Longman, Singapore).
23. Jha AK, Freed KF (2008) Solvation effect on conformations of 1,2:dimethoxyethane: Charge-dependent nonlinear response in implicit solvent models. *J Chem Phys* 128:034501.
24. Verdier PH, Stockmayer WH (1962) Monte Carlo calculations on dynamics of polymers in dilute solution. *J Chem Phys* 36:227-235.
25. Lal M (1969) Monte Carlo computer simulation of chain molecules. I. *Molec Phys* 17:57-64.
26. Metropolis N, Rosenbluth AW, Rosenbluth MN, Teller E (1953) Equation of state calculations by fast computing machines. *J Chem Phys* 21:1087–1092.
27. Li KKC, Lee KAW (2000) Transcriptional activation by the EWS oncogene can be cis-repressed by the EWS RNA-binding domain. *J Biol Chem* 275:23053-23058.
28. Alex D, Lee KAW (2005) RGG-boxes of the EWS oncoprotein repress a range of transcriptional activation domains. *Nucl Acids* Res 3:1323-1331.
29. Borg M, Mittag T, Pawson T, Tyers M, Forman-Kay JD, Chan HS (2007) Polyelectrostatic interactions of disordered ligands suggest a physical basis for ultrasensitivity. *Proc Natl Acad Sci USA* 104:9650-9655.
30. Domb C (1969) Self-avoiding walks on lattices. *Adv Chem Phys* 15:229-259.
31. Chan HS, Dill KA (1990). The effects of internal constraints on the configurations of chain molecules. *J Chem Phys* 92:3118-3135.
32. Chan HS, Dill KA (1991) Polymer principles in protein structure and stability. *Annu Rev Biophys Biophys Chem* 20:447-490.
33. Chan HS, Wattenbarger MR, Evans DF, Bloomfield VA, Dill KA (1991) Enhanced structure in polymers at interfaces. *J Chem Phys* 94:8542-8557.
34. Sykes MF (1963) Self-avoiding walks on the simple cubic lattice. *J Chem Phys* 39:410-412.
35. Clisby N, Liang R, Slade G (2007) Self-avoiding walk enumeration via the lace expansion. *J Phys A: Math Theor* 40:10973-11017.
36. Dill KA, Alonso DOV (1988) Conformational entropy and protein stability. In *Protein Structure and Protein Engineering*, 39, Huber R, Winnacker EL, eds. *Colloquium-Mosbach der Gesellschaft fur Biologische Chemie* (Berlin: Springer-Verlag), pp.51-58.
37. Marshall MS, Steele RP, Thanthiriwatte, Sherrill CD (2009) Potential energy curves for cation-π interactions: off-axis configurations are also attractive. *J Phys Chem A* 113:13628-13632.

Supporting Figures

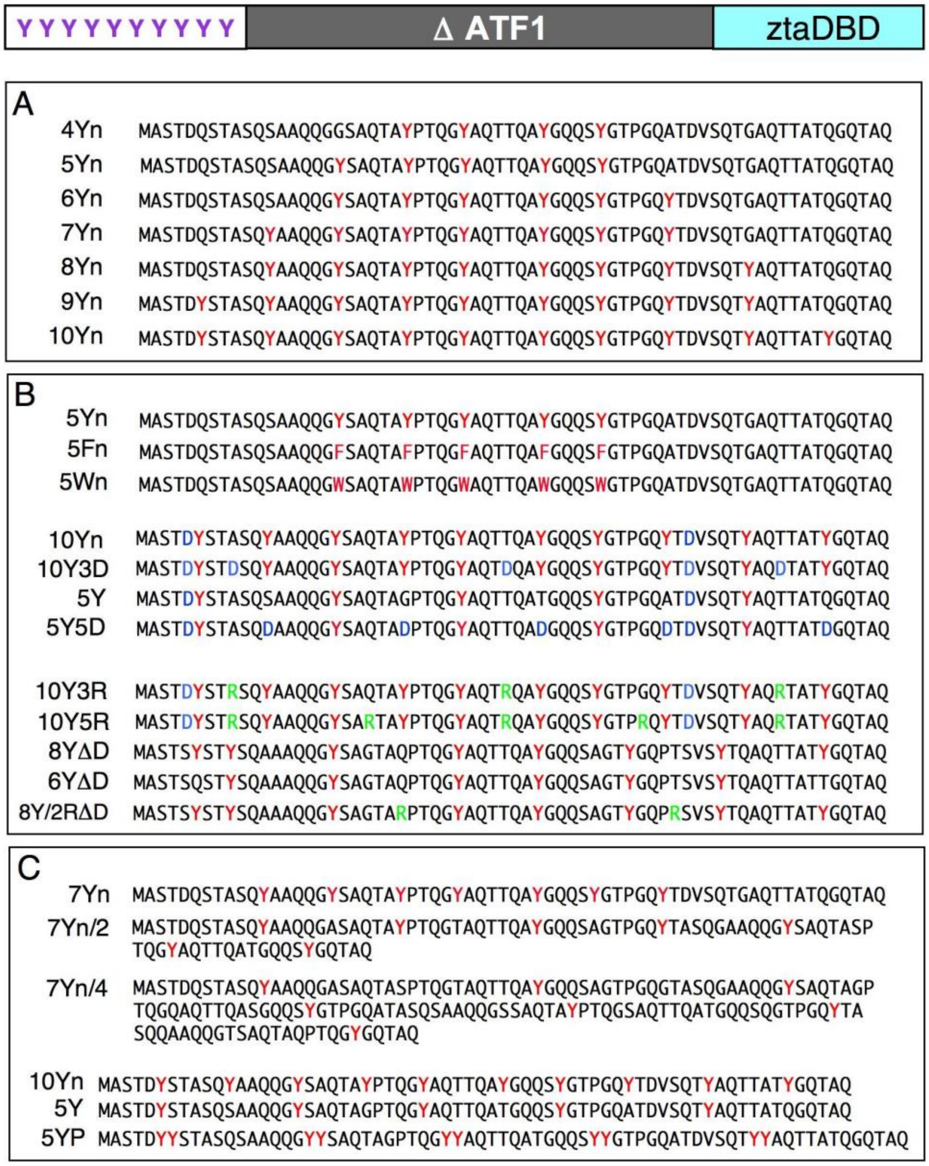

Figure S1. Proteins and EAD sequences used in the present study. Transcriptional activator proteins (*Top*) contain the experimental sequences related to the N-terminal 66 residues of EAD1-66 (box with purple Ys), the region of ATF1 protein (ΔATF1) present in the EWS/ATF1 oncogene and the DNA-binding domain of zta protein (ztaDBD). In (A)–(C), amino acid residues are denoted by the standard one-letter code. Sequences for Figs. 1, 3, and 4 in the main text are listed, respectively, under (A), (B) and (C).

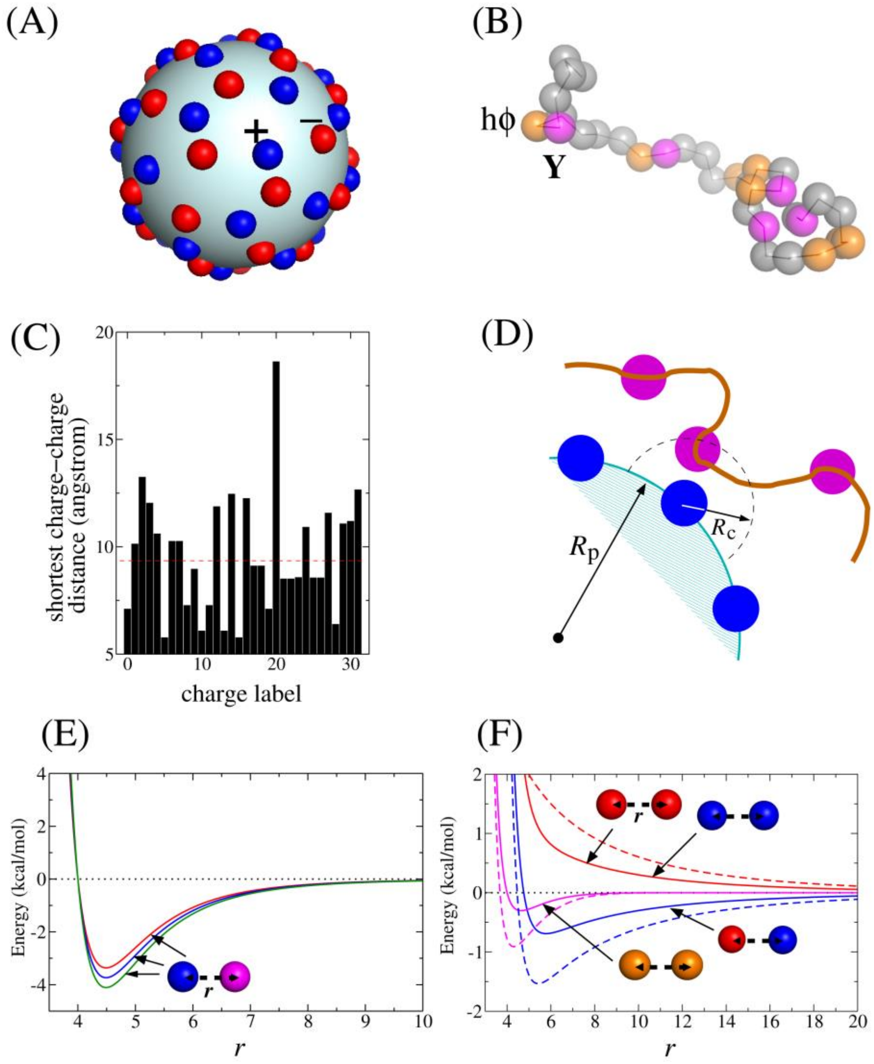

Figure S2. The chain simulation model. (A) The generic EAD binding target (partner) is a sphere of radius R_p = 16Å with essentially evenly distributed positive and negative charges (represented by blue and red beads respectively). (B) An EAD sequence is modeled as a C_α chain (beads on a string) that can engage in cation-π, electrostatic, hydrophobic, and excluded-volume interactions as specified in the main text and Text S1. In this figure and subsequent supporting figures, aromatic (Y in this drawing) and hydrophobic (hφ) residues are shown in magenta and orange, respectively, whereas positively and negatively charged residues are shown in blue and red respectively. All other residues are shown in grey. (C) The distribution of positively charged residues on the heterodimer of the Rpb4/Rpb7 subunits of human RNA polymerase II was used as a reference for the design of the charge density on the generic EAD binding target. The histogram here shows the shortest distance from each of the 32 positively charged amino acid residues (R or K) on Rpb4/Rpb7 (16 each along the Rpb4 and Rpb7 chains) from another positively charged residue, based on the X-ray crystal structure (PDB ID: 2C35) determined by Meka et al. (ref. [10] of Text S1). The distances are measured between the atoms that have the positive charges. The red dashed horizontal line marks the average shortest distance which is ≈ 9.4Å. (D) EAD-target binding is defined in the model as having at least one EAD aromatic residue (magenta circle) within a capture radius R_c = 6Å from a positive charge (blue circle) on the target. One such cation-π contact between an EAD sequence (brown string connecting magenta circles) and the target (large shaded circle with embedded blue circles) is shown in this schematic

drawing. (E,F) Energetic components of the interaction potential, the horizontal variable r here corresponds to r_{ij} in Eq. (**S1**) or $r_{i\nu}$ in Eq. (**S2**). (E) Model cation-π interaction potentials in the form of $\varepsilon_{\mathrm{c}\pi}^{ij}\left[\left(\sigma_{\mathrm{c}\pi}/r_{ij}\right)^{12} - \left(\sigma_{\mathrm{c}\pi}/r_{ij}\right)^{6}\right]$ or $\varepsilon_{\mathrm{c}\pi}^{i\nu}\left[\left(\sigma_{\mathrm{c}\pi}/r_{ij}\right)^{12} - \left(\sigma_{\mathrm{c}\pi}/r_{ij}\right)^{6}\right]$ in Eqs. (**S1**) and (**S2**) respectively [i.e., equivalent to Fig. 1B in the main text minus the $\varepsilon_{\mathrm{ex}}(r_{\mathrm{rep},ij}/r_{ij})^{12}$ term]. The green and blue curves show the potentials for cation-W and cation-Y, respectively, as in Fig. 1B, whereas the red curve corresponds to the weakest among the model cation-F interactions considered in Fig. 1B. (F) Total interaction potential between hydrophobic residues and between charged residues in the simulation chain model, including their respective excluded-volume interactions. Solid curves show potential functions used for all simulation results presented in this work except specifically noted otherwise. Dashed curves show alternative potential functions that we have used for the control simulations reported in Text S1. The potential functions used for hydrophobic interaction are shown in magenta. The solid curve is for hydrophobic interaction strength $\varepsilon_{\mathrm{h}\varphi}$ = –3.0 $k_{\mathrm{B}}T$ [Eq. (**S1**)] whereas the dashed curve is for $\varepsilon_{\mathrm{h}\varphi}$ = –7.0 $k_{\mathrm{B}}T$. The potential functions for electrostatic interactions between like charges and between opposite charges are shown, respectively, in red and blue. The solid curves are for ϵ_{d} = 40 whereas the dashed curves are for ϵ_{d} = 20.

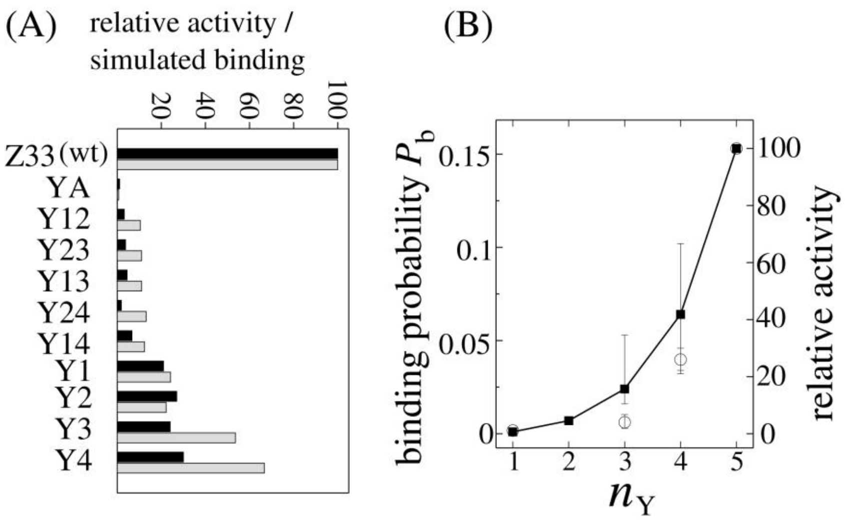

Figure S3. Evidence for the polycation-π hypothesis from a re-analysis of early experiments on 33-residue EAD sequences. Sequences and experimental data were taken from ref. [1] of Text S1. Simulations were conducted using the same chain model as described in Text S1 and the main text in a (600Å)^3 simulation box. (A) The sequences are defined in the above reference. The experimental relative activities and the simulated relative binding probabilities are represented by the black and grey bars respectively. (B) The sequences in (A) are grouped according to their Y number n_{Y}. Plotted are the simulated binding probability (solid squares) and the relative experimental activity (open circles) averaged over sequences belonging to each given n_{Y}. For the simulation results, the averages are over all possible permutations of Y positions for a given n_{Y}, including those not studied by experiments. Note that both Y number and Y density are varied among this set of sequences (unlike the set in Fig. 1 that varies only the Y number while keeping Y density constant). Error bars show variation among sequences with the same n_{Y}. Lines joining the solid squares are merely a guide for the eye.

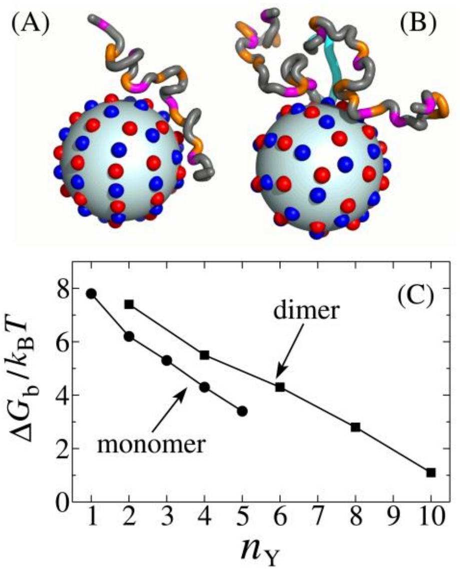

Figure S4. Simulated binding probabilities of monomer and dimer EAD sequences follow similar trends. Similar dependences on n_Y are observed for cis-duplication of small EAD elements in a single dimer. The monomer sequences used in the present simulations are the same 33-residue sequences based on the construction by Feng and Lee (ref. [1] of Text S1) studied in Fig. S3. As for the simulations in Fig. S3, all possible permutations of Y positions are considered. Each dimer was constructed by joining the C-terminus of a given monomer sequence to the C-terminus of another copy of the same monomer sequence by a linker chain. The linker consists of six residues that are neither charged nor hydrophobic; all reference bond angles within the linker are equal to 165 °with a stiff bond-angle force constant equal to $10.0k_BT$. Thus, in this figure, a dimer sequence with Y number $2n_Y$ is equivalent to two identical monomer sequences with Y number n_Y connected by such a linker. (A) A snapshot of an $n_Y = 5$ monomer bound to the target. (B) A snapshot of the corresponding $n_Y = 10$ dimer bound to the target. The EAD chains are depicted in a tube representation with the color code for different residue types specified in Fig. S2B. (C) Free energies of binding were computed under the same conditions as those used for Fig. S3. ΔG_b values averaging over sequences with the same n_Y are plotted.

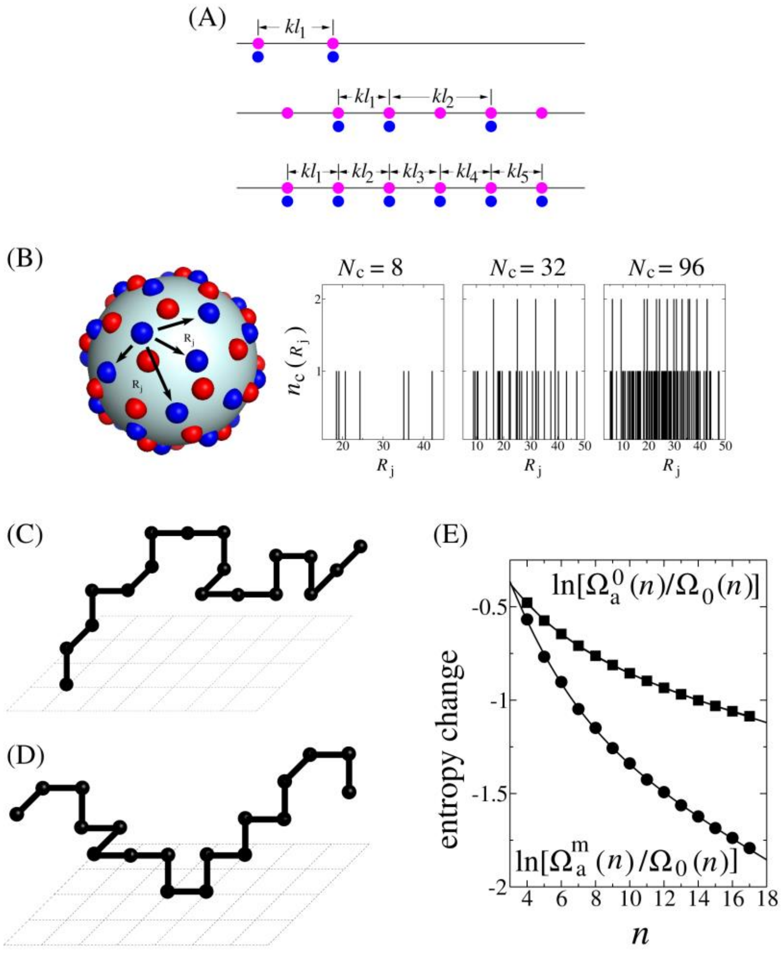

Figure S5. Components of the analytical model. (A) Schematic of cation-π contacts along the IDP. Here we only consider IDP chains with evenly spaced aromatics that are k residues apart; thus the contour length between two cation-contacting aromatics is always in the form of kl_i where l_i is a positive integer. Three example contact patterns are shown, wherein the aromatics and cations are depicted as magenta and blue circles respectively. (B) Distribution of cation-cation distance R_j on the target. Each R_j value is the distance in Å from a given cation to a different cation, measured on the spherical surface of the model target (left drawing). The distribution $n_c(R_j)$ is shown (histograms) for three different targets of the same size but different cation densities. As for the target with N_c = 32 cations in most of our simulations, the cations are essentially evenly distributed on the surface for the N_c = 8 and N_c = 96 targets. The approximately even distribution of charges on the target sphere was achieved by a numerical algorithm (see Text S1). As can be seen from the histograms, only a few of the R_j values are exactly identical. (C) An example conformation configured in the simple cubic lattice with one end of the chain touching a plane. The number of such conformations is referred to as $\Omega_a^0(n)$ in this work. (D) An example simple cubic lattice conformation with two of its mid-chain sites in contact with a plane. We denote the number of such conformations as $\Omega_a^m(n)$. (E) Change in conformational entropy (in units of the Boltzmann constant k_B) upon bringing a free lattice conformation to form a contact at a chain end (squares) or at mid-chain (circles) with an infinite impenetrable plane that imposes excluded volume on the other side of the plane (the space underneath the plane is not accessible to the chain). The data points (squares or circles) were computed using exact enumeration data in Table S1. The curves through the data points were generated by fitting the assumed relation $y = \ln[A\exp(-\omega n) + B\exp(-\sigma n)]$. The fitting parameters here are A = 0.5365, B = 0.53139, ω = 0.02786, and σ = 0.33604 for $y = \ln[\Omega_a^0(n)/\Omega_0(n)]$; and A = 0.40915, B = 1.12627, ω = 0.05373, and σ = 0.39353 for $y = \ln[\Omega_a^m(n)/\Omega_0(n)]$.

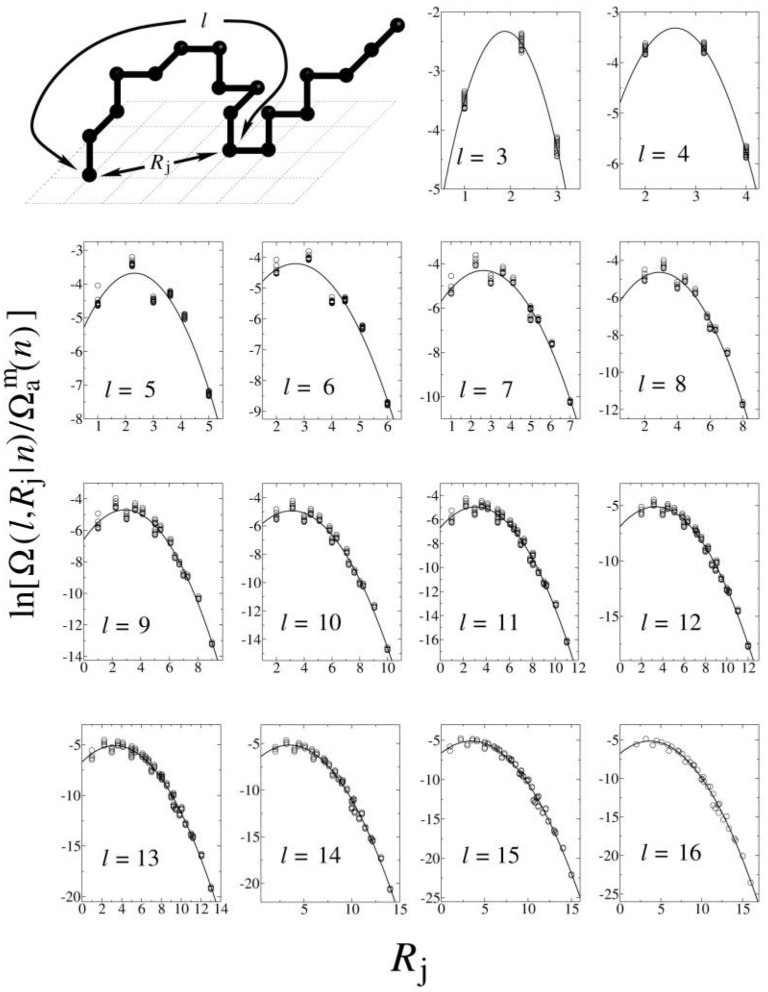

Figure S6. Conformational entropy loss upon loop formation. The quantity $\Omega(l, R_j|n)$ is the number of simple cubic lattice conformations of length *n* (*n* is the total number of beads along the chain) that have one chain end (bead number 1) touching an excluded-volume plane at a given point (as in Fig. S5C) and, at the same time, bead number *l* + 1 also making a contact with a given point on the plane at a distance R_j from where bead number 1 touches the plane, thus forming a loop of length *l* that spans a distance R_j on the plane (top left drawing). Note that conformations that form other chain-plane contact(s) in addition to these two are included in the $\Omega(l, R_j|n)$ count. As discussed in the main text and in Text S1, the vertical variable $\ln[\Omega(l, R_j|n)/\Omega_a^m(n)]$ for the plots in this figure corresponds approximately to the conformational entropy change, in units of k_B, upon making an additional chain-plane contact to form a loop of length *l* along a chain that has already made at least one contact with the plane. Each of the plotting panels provides the conformational entropy change upon forming a loop of a given length *l* as a function of R_j. Both *l* and R_j are shown in units of the lattice bond length (nearest distance between two beads on the simple cubic lattice). Data points (open circles) in the plotting panels were computed by exact enumeration of lattice conformations with chain lengths from *n* = 4 through *n* = 17 (see Text S1 and Tables S2 and S3). Multiple data points for the same R_j value represent results from different *n* values. The continuous curves are quadratic fits in the form of $\ln[\Omega(l, R_j|n)/\Omega_a^m(n)] = -a(l)[R_j - b(l)]^2 + c(l)$. The *l*-dependent fitting parameters $a(l)$, $b(l)$, and $c(l)$ are provided in Fig. S7. In view of the clustering of data points from different *n* values, we have made an approximation in the analytical model that $\ln[\Omega(l, R_j|n)/\Omega_a^m(n)]$ is independent of *n*.

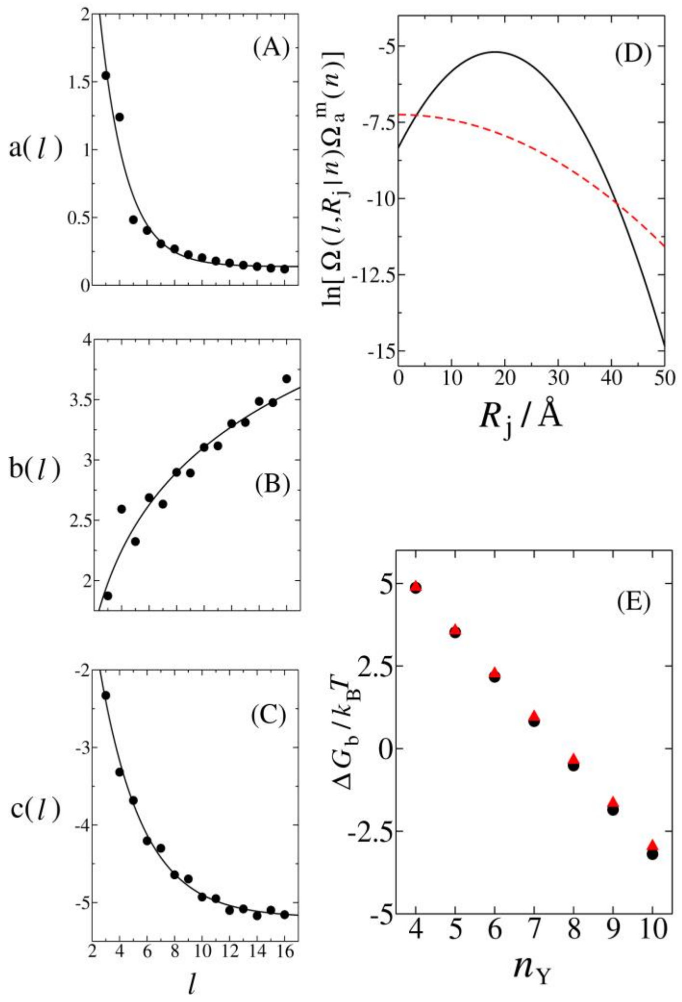

Figure S7. Applying the lattice conformational entropy estimates to the analytical model. (A–C) The fitting parameters $a(l)$, $b(l)$, and $c(l)$ for the conformational entropy changes shown in Fig. S6 are provided as data points in (A), (B), and (C), respectively. The continuous fitting curves are given by (A) $a(l) = A + B\exp(-Cl)$, where A = 0.13748, B = 7.04181, and C = 0.52115; (B) $b(l) = A + B\ln(Cl)$, where A = 0.97499, B = 0.93564, and C = 0.97495; and (C) $c(l) = A + B\exp[-C(l-D)]$, where A = –5.19530, B = 2.98286, C = 0.31975, and D = 2.79004. These expressions were used to estimate $\ln\left[\Omega\left(l, R_j|n\right)/\Omega_{\rm a}^{\rm m}(n)\right]$ for l > 16 by extrapolation. (D) The extrapolated $\ln\left[\Omega\left(l, R_j|n\right)/\Omega_{\rm a}^{\rm m}(n)\right]$ function (black curve) is compared against the corresponding random-flight expression $\ln\left[(3/2\pi l)^{3/2}\exp\left(-3R_j^2/2l\right)\right]$ (red dashed curve) for l = 60. (E) Two methods for estimating the entropic cost of loop formation in the analytical model are compared. Plotted are the binding free energies of the model EAD chains in Fig. 1 for $E_{{\rm c}\pi} = -3.5k_{\rm B}T$. The black data points (circles) were computed by using entropy estimates from exact enumeration for $l \leq 16$ and extrapolated estimates for l > 16, whereas the red data points (triangles) were obtained by using entropy estimates from exact enumeration for $l \leq 16$ but random-flight estimates for l > 16. The plot here shows that the predicted $\Delta G_{\rm b}$ values based on the two different loop entropy estimates are very similar.

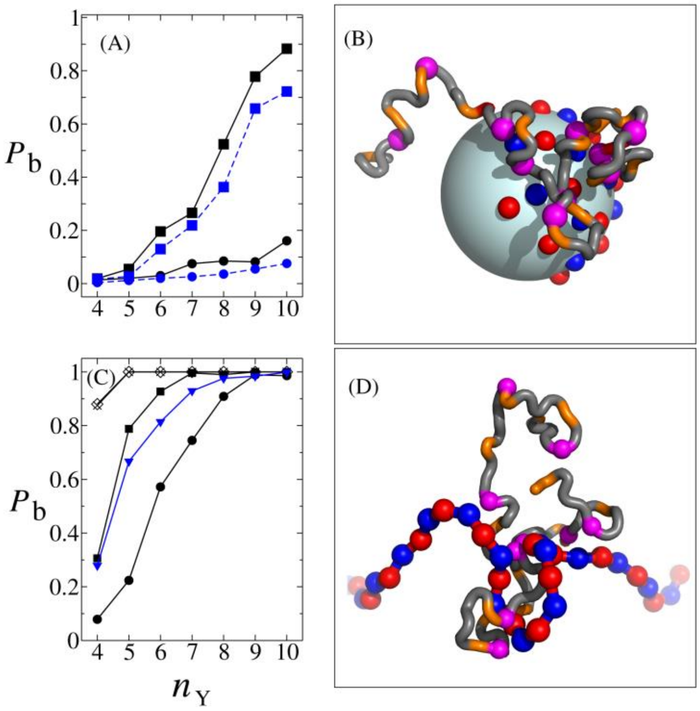

Figure S8. Exploring other EAD-target binding scenarios. The EAD sequences are the same as those in Fig. 1. (A) Simulated EAD binding probability P_b with a hypothetical target in which the surface charges are not evenly distributed but confined to a patch. Two such hypothetical patch partners were considered, both with 12 cations localized on a patch with the same local cation density as the generic target with 32 cations (Fig. S2A) that we have used for most of the simulations. One of the targets (referred to as the positive patch target) contains 12 cations and no anions on the patch whereas the other (referred to as the neutral patch target) contains 12 cations and 12 anions. Plotted here are the simulated binding probabilities for the positive (squares) and neutral (circles) patch targets in either a simulation box of size of $(300Å)^3$ (black symbols) or $(600Å)^3$ (blue symbols). (B) A snapshot of an $n_Y = 10$ EAD sequence (tube representation) bound to the neutral patch target. (C) Simulated EAD binding probability P_b with hypothetical disordered (IDP) partners. The EAD sequences and simulation conditions are the same as those in Fig. 1B,C, using a simulation box of size $(600Å)^3$. During the binding simulations, both the EAD and the hypothetical IDP target were allowed to sample all accessible conformations while the center of mass of the IDP target was kept at a fixed position in the center of the simulation box. We considered a class of such targets, each of which is a chain consisting of 64 alternating cations and anions (32 cations and 32 anions). The adjacent cation and anion are connected by a 5Å virtual bond with a stiff bond-angle force constant equal to $10.0k_BT$. Shown here are binding probabilities for four different such IDP targets with equilibrium bond angles that equal, respectively, to 105 °(crosses), 120 °(diamonds), 135 °(squares) and 150 °(circles). A general trend of increasing binding with increasing n_Y is observed for all four hypothetical IDP targets. Not surprisingly, the quantitative details of this trend are sensitive to the persistence length of the IDP target. Binding increases with the flexibility of the IDP target. Also included for comparison (blue triangles) are the simulated probabilities of EAD binding with the RGG3 sequence in the Ewing's sarcoma RNA-binding domain GGDRGRGGPGGMRGGRGGLMDRGGPGGMFRGGRGGDRGGFRGGRGMDRGGFGGGRRGGPGG (refs. [27,28] in Text S1). Here the RGG3 sequence was modeled as a C_α chain using the same modeling scheme as that for the EAD sequences. (D) A snapshot of an $n_Y = 10$ EAD sequence (tube representation) bound to a hypothetical IDP target (red and blue beads) with 150 °bond angles.

Supporting Tables

n	$\Omega_0(n)$	$\Omega_a^0(n)$	$\Omega_a^m(n)$
4	150	93	85
5	726	409	337
6	3,534	1,853	1,433
7	16,926	8,333	5,937
8	81,390	37,965	25,809
9	387,966	172,265	110,369
10	1,853,886	787,557	486,049
11	8,809,878	3,593,465	2,118,369
12	41,934,150	16,477,845	9,427,777
13	198,842,742	75,481,105	41,662,809
14	943,974,510	346,960,613	186,303,561
15	4,468,911,678	1,593,924,045	828,799,641
16	21,175,146,054	7,341,070,889	3,725,715,541
17	100,121,875,974	33,798,930,541	16,682,103,329

Table S1. Numbers of conformations, or self-avoiding flights, on the simple cubic lattice. Conformational counts as functions of chain length (number of beads) n are obtained by exact enumeration. A chain with n beads has n – 1 bonds. Here, Ω_0 is the number of unconstrained conformations; Ω_a^0 is the number of conformations that have one chain end anchored onto an impenetrable plane (Fig. S5C); and Ω_a^m is the number of conformations that have the mid-chain bead [$(n/2)^{th}$ bead if n is even, $\{(n+1)/2\}^{th}$ bead if n is odd] making a contact with an impenetrable plane (Fig. S5D).

x,y	R_j	$l = 3$			$l = 4$		$l = 5$
		$n = 4$	$n = 5$	$n = 6$	$n = 5$	$n = 6$	$n = 6$
0,1	1	3	9	38			
0,3	3	1	4	17			
1,2	$\sqrt{5}$	6	24	98			
0,2	2				9	33	
0,4	4				1	4	
1,3	$\sqrt{10}$				8	32	
0,1	1						25
0,3	3						18
0,5	5						1
1,2	$\sqrt{5}$						58
1,4	$\sqrt{17}$						10
2,3	$\sqrt{13}$						20

Table S2. Loop probabilities determined by exact lattice conformational enumeration. Tabulated here are examples (not a complete list) of conformational counts $\Omega(l, R_j|n)$ used in Fig. S6. Here one chain end is always in contact with the origin (0,0) of a two-dimensional coordinate system for the impenetrable plane. In this table, the positions on the impenetrable plane where another contact with the chain existed are indicated by the (x,y) coordinates. In the present treatment of our analytical model, R_j values from all combinations of x,y (where $x < y$) that have nonzero $\Omega(l, R_j|n)$ counts for $n \leq 17$ were used to estimate the conformational entropic cost of loop formation (Figs. S6 and S7).

	0	1	2	3	4	5	6	7	8	9	10	11	12	13	14	15	16
0	0	0	62,068,495	0	56,801,564	0	27,780,947	0	6,593,054	0	659,638	0	25,006	0	315	0	1
1		13,944,500	0	128,368,232	0	83,248,956	0	28,370,408	0	4,339,452	0	261,696	0	5,492	0	32	0
2			131,632,780	0	102,360,344	0	46,404,240	0	10,178,096	0	911,232	0	28,658	0	240	0	0
3				108,903,312	0	60,853,620	0	18,005,904	0	2,278,472	0	101,192	0	1,120	0	0	0
4					66,318,904	0	24,958,578	0	4,275,524	0	257,894	0	3,640	0	0	0	0
5						27,752,872	0	6,179,844	0	492,492	0	8,736	0	0	0	0	0
6							6,976,992	0	720,434	0	16,016	0	0	0	0	0	0
7								816,816	0	22,880	0	0	0	0	0	0	0
8									25,740	0	0	0	0	0	0	0	0
9										0	0	0	0	0	0	0	0
10											0	0	0	0	0	0	0
11												0	0	0	0	0	0
12													0	0	0	0	0
13														0	0	0	0
14															0	0	0
15																0	0
16																	0

Table S3. Exact lattice enumeration data for loop formation probability. Tabulated here as examples are the exact $\Omega(l, R_j|n)$ counts for l = 16 and n = 17. The horizontal and vertical labels correspond, respectively, to the x and y coordinates of the positions on the impenetrable plane. One end of the chain (first bead) is always anchored at the origin (0,0). In this table, the entry at a given position (x,y) is the number of conformations that have the chain's last (n^{th}) bead contacting the given position and thus making a loop with $R_j = \sqrt{x^2 + y^2}$. Data are shown only for $x \leq y$ because of the obvious rotational symmetry.
